# Gene co-expression network analysis of *Trypanosoma brucei* in tsetse fly vector

**DOI:** 10.1186/s13071-021-04597-6

**Published:** 2021-01-22

**Authors:** Kennedy W. Mwangi, Rosaline W. Macharia, Joel L. Bargul

**Affiliations:** 1 International Centre of Insect Physiology and Ecology (icipe), P.O. Box 30772-00100, Nairobi, Kenya; 2 Jomo Kenyatta University of Agriculture and Technology, P.O. BOX 62000-00200, Nairobi, Kenya; 3 University of Nairobi, P.O. Box 30197-00100, Nairobi, Kenya

**Keywords:** *Trypanosoma brucei*, Tsetse fly, Gene co-expression network, Weighted gene co-expression network analysis

## Abstract

**Background:**

*Trypanosoma brucei* species are motile protozoan parasites that are cyclically transmitted by tsetse fly (genus *Glossina*) causing human sleeping sickness and nagana in livestock in sub-Saharan Africa. African trypanosomes display digenetic life cycle stages in the tsetse fly vector and in their mammalian host. Experimental work on insect-stage trypanosomes is challenging because of the difficulty in setting up successful *in vitro* cultures. Therefore, there is limited knowledge on the trypanosome biology during its development in the tsetse fly. Consequently, this limits the development of new strategies for blocking parasite transmission in the tsetse fly.

**Methods:**

In this study, RNA-Seq data of insect-stage trypanosomes were used to construct a *T. brucei* gene co-expression network using the weighted gene co-expression analysis (WGCNA) method. The study identified significant enriched modules for genes that play key roles during the parasite’s development in tsetse fly. Furthermore, potential 3′ untranslated region (UTR) regulatory elements for genes that clustered in the same module were identified using the Finding Informative Regulatory Elements (FIRE) tool.

**Results:**

A fraction of gene modules (12 out of 27 modules) in the constructed network were found to be enriched in functional roles associated with the cell division, protein biosynthesis, mitochondrion, and cell surface. Additionally, 12 hub genes encoding proteins such as RNA-binding protein 6 (RBP6), arginine kinase 1 (AK1), *brucei* alanine-rich protein (BARP), among others, were identified for the 12 significantly enriched gene modules. In addition, the potential regulatory elements located in the 3′ untranslated regions of genes within the same module were predicted.

**Conclusions:**

The constructed gene co-expression network provides a useful resource for network-based data mining to identify candidate genes for functional studies. This will enhance understanding of the molecular mechanisms that underlie important biological processes during parasite’s development in tsetse fly. Ultimately, these findings will be key in the identification of potential molecular targets for disease control.
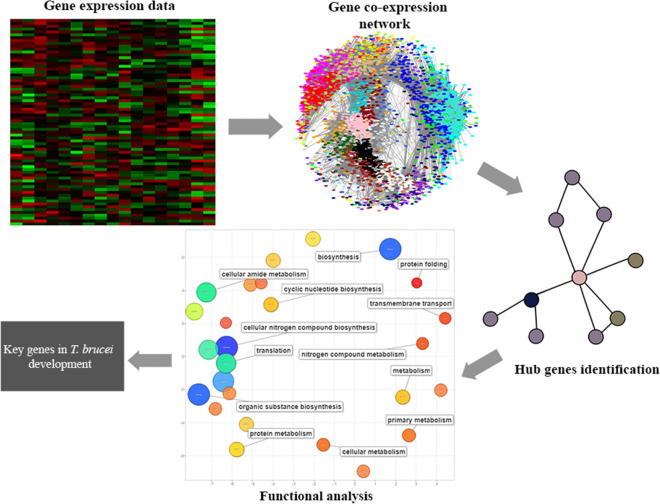

## Background

*Trypanosoma brucei* has a digenetic life cycle with distinct morphological forms existing during its development in the mammalian host and the tsetse fly [1]. In the mammalian bloodstream, the morphological forms are slender and stumpy trypomastigotes, while in the tsetse fly, they comprise procyclic trypomastigote forms in the midgut, long and short epimastigotes in the proventriculus, and short epimastigote and metacyclic trypomastigotes in the salivary glands [2, 3]. Most *T. brucei* research has focused on the mammalian bloodstream and tsetse procyclic forms of trypanosomes as they are relatively easier to maintain in *in vitro* cultures [4, 5]. Consequently, this has led to less exploration of parasite phenotypes in the tsetse fly that could provide insights into the biology of a trypanosome during its development in the vector—the life cycle phase referred to as “the heart of darkness” [6]. The knowledge of trypanosome development in the tsetse fly will contribute to efforts towards interrupting disease transmission by the vector. This can be achieved through targeted disruption of the parasite’s essential molecular processes such as motility, regulation of differentiation, morphological remodeling, and signal transduction [7, 8].

In the last decade, RNA-Seq technology has been a fundamental tool for studying gene expression profiles of *T. brucei* and other kinetoplastids with an aim of expanding knowledge on their biology [9]. This is because RNA-Seq provides a comprehensive and more accurate transcriptome quantification and characterization compared to the hybridization-based techniques such as microarray [10]. In addition to identification of differentially expressed genes, transcriptome data could also be used to create gene co-expression networks which provide a functional and molecular understanding of key biological processes in an organism [11, 12].

Gene co-expression network analysis aims to identify coordinated gene expression patterns that indicate functional relationships between the expressed genes. Using a method such as WGCNA [13], highly correlated genes are grouped into modules (gene clusters) which are currently thought to be co-expressed and hence perform similar biological functions [14]. Each module is believed to encode a specific biological function based on the genes it contains. To associate genes in a given module to specific functions, an enrichment analysis is performed against databases such as gene ontology (GO) [15] and Kyoto Encyclopedia for Genes and Genomes (KEGG) [16].

Furthermore, WGCNA allows identification of intra-modular hub genes, which are usually the highly connected genes in a module [13, 17]. These hub genes could play key roles in the biological functions of their modules or act as representatives of their predominant biological function [11]. Also, based on the hypothesis that functionally related genes may be co-regulated, co-expression network modules are useful in gene regulation analysis including prediction of regulatory elements (motifs) for genes in the same module [18, 19]. Additionally, functions of uncharacterized genes are predicted based on their co-expression with genes of known function in the co-expression network, a principle referred to as “guilt by association” [12].

The present study aimed at generating a gene co-expression network to explore functionally relevant genes involved in *T. brucei* development in the tsetse fly vector. In contrast to a *T. brucei* gene co-expression network generated from a previous study for procyclic and bloodstream forms using microarray data [20], our study focused on the insect stage morphological forms of the parasite by analyzing RNA-Seq data. The constructed gene co-expression network permitted identification of 12 functionally relevant modules and their 12 hub genes as well as potential regulatory motifs in the mRNA’s 3′ untranslated regions for genes grouped in the same module.

## Methods

### Datasets acquisition and quality assessment

RNA-Seq datasets of *Glossina morsitans morsitans* (tsetse fly) trypanosome-infected midgut, proventriculus, and salivary gland tissues were obtained from the European Nucleotide Archive (ENA) [21] under accession numbers SRP002243 and SRR965341. The dataset consisted of 18 samples: 7 midgut; 4 proventriculus; 7 salivary glands [22, 23]. The quality of the data was assessed using FastQC version 0.11.8 (http://www.bioinformatics.babraham.ac.uk/projects/fastqc/). Prior to reads mapping, the *T. brucei* genome and *G. morsitans* scaffold genome were obtained from TriTrypDB (Release 43) [24] and VectorBase [25], respectively, and concatenated to create a chimeric genome. The RNA-Seq reads were mapped to the chimeric genome of *T. brucei* and *G. morsitans* using HISAT2 version 2.1.0 [26] to remove ambiguously mapped reads. Duplication rates were computed after read mapping using the MarkDuplicates tool from Picard toolkit version 2.20.3 (http://broadinstitute.github.io/picard/) to mark duplicate reads. Furthermore, dupRadar Bioconductor R package version 1.18.0 was used to assess the RNA-Seq data for presence of PCR duplicates [27]. Samples that had PCR duplicates were excluded from downstream analysis.

### Reads quantification

The reads that mapped to *T. brucei* genome were counted using HTSeq version 0.11.2 [28] and in relation to the annotation file of *T. brucei* downloaded from TriTrypDB (Release 43). Non-protein coding genes (ncRNA, snRNA, snoRNA, pseudogenic transcripts, rRNA, and tRNA) were excluded from the read counts as this study focused on protein-coding genes and their functional analysis.

### Sample quality assessment and filtering

Genes with low expression levels were removed from the read counts data using *filterByExpr* function from R package edgeR version 3.8 [29]. Sample quality was assessed using Pearson correlation heatmaps and Principal Component Analysis (PCA) and box plots in R version 3.6.0 [30]. Trimmed mean of M-values (TMM) was used as a normalization method using *calcNormFactors* function in edgeR [29]. The normalized read counts were then converted to counts per million and log_2_ transformed for downstream analysis. Batch effects were adjusted for using the ComBat method from sva R package version 3.32.1 [31].

### Construction of the weighted gene co-expression network

The weighted gene co-expression network was constructed using WGCNA R package version 1.66 [17]. First, soft-thresholding power, β, was determined using the *pickSoftThreshold* function from WGCNA package. This was followed by the construction of a weighted adjacency matrix using the *adjacency* function, after which the matrix was computed into the Topological Overlap Matrix (TOM) using the *TOMsimilarity* function [13]. The TOM measure between pairs of genes was used as input for average linkage hierarchical clustering by first creating a dissimilarity matrix (dissTOM = 1 − TOM) and then using the *flashClust* function to create the gene tree dendrogram. The Dynamic Tree Cut algorithm was used to identify modules using the gene tree dendrogram as input for the *cutreeDynamicTree* function from dynamicTreeCut R package version 1.63-1 [32]. The *chooseTopHubInEachModule* function from the WGCNA package was used to identify the hub genes.

### Network functional enrichment analysis and visualization

The goseq R package version 1.36.0 [33] was used to test for enrichment of gene ontology (GO) [15] and Kyoto Encyclopedia of Gene and Genomes (KEGG) [16] annotations for each of the identified modules. The GO and KEGG annotations were obtained from TriTrypDB. The generated lists of GO terms for the modules were summarized using REVIGO (http://revigo.irb.hr/) [34]. Cytoscape version 3.7.1 [35] was used to visualize the network using the *exportNetworkToCytoscape* function from the WGCNA package.

### Prediction of 3′ UTR regulatory motifs

All the genes in the gene co-expression network and their corresponding cluster/module index were used to generate an expression file that was used as input for the tool FIRE, version 1.1a [19]. This expression file was submitted online to FIRE (https://tavazoielab.c2b2.columbia.edu/FIRE/) with default parameters for prediction of 3′ UTR motifs.

The code used in data pre-processing, network construction, and functional analysis is provided as Additional file 1 and archived at: https://github.com/wanjauk/tbrucei_gcn. Motif prediction was performed online at: https://tavazoielab.c2b2.columbia.edu/FIRE/.

## Results

### Data pre-processing

A total of 18 samples of raw RNA-Seq data (Additional file 2: Table S1) were obtained for this study. Of the 18 samples, 3 generated from the trypanosome-infected salivary glands were excluded from further analysis because they contained PCR duplicates. Thus, a total of 15 samples were analyzed (Additional file 2: Table S1). Furthermore, lowly expressed genes were excluded to reduce noise, thus resulting in a total of 7390 genes across the 15 samples.

**Fig. 1:**
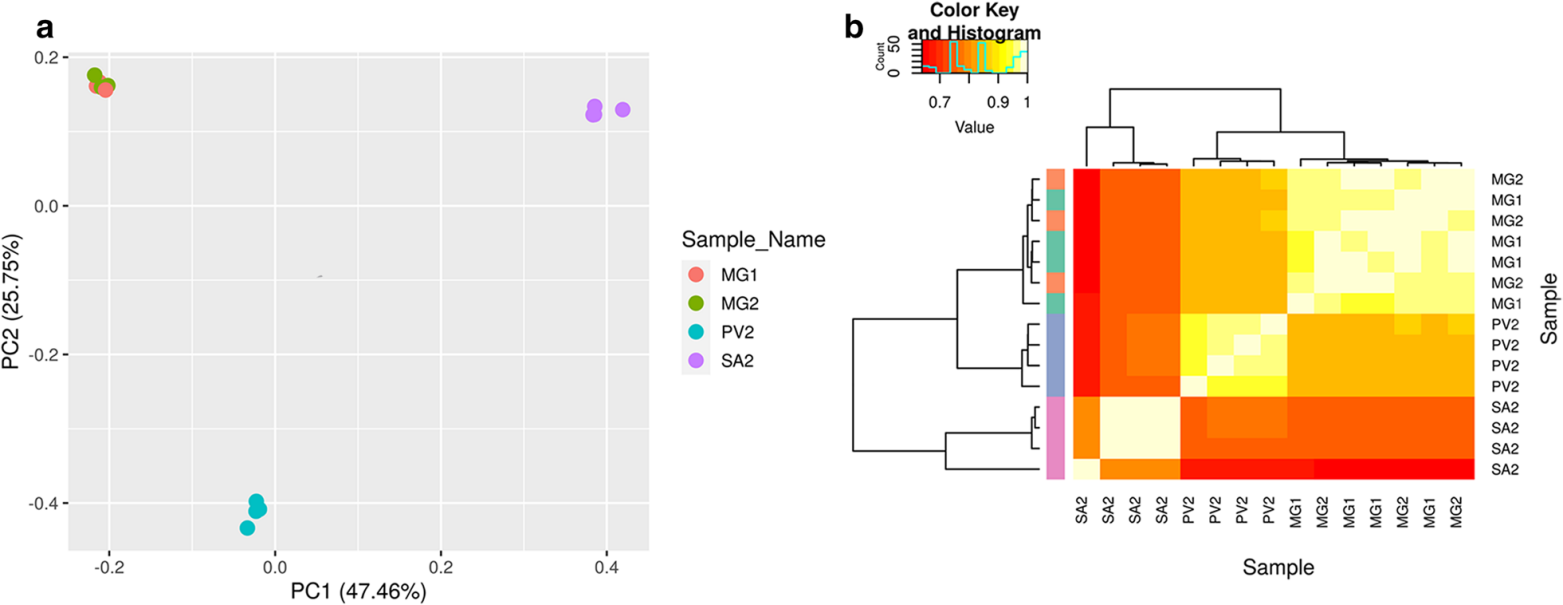
Global gene expression profiles of *Trypanosoma brucei*. **a** Principal component analysis (PCA) plot. Each point in the PCA plot represents a sample, and point color indicates a batch that consists of the biological replicates. **b** Sample correlation heatmap using hierarchical clustering. Color codes along the left side of the sample correlation heatmap indicate samples based on the batch they belong to. MG1 and MG2 are midgut samples, PV2 proventriculus samples, and SA2 salivary gland samples

The relationship between the samples and the reproducibility of biological replicates was determined using principal component analysis (PCA) and Pearson correlation heatmap analysis prior to (Additional file 3: Figure S1) and after adjusting for batch effects that could have resulted from biological replicates (Fig. 1). The PCA and Pearson correlation heatmap plots showed that the samples grouped together based on the developmental stages of *T. brucei* in the insect vector rather than their biological replicates (Fig. 1). An assessment of the distribution of per-gene read counts per sample showed a median steady-state expression level of ~ 6.5 log_2_ counts per million in all the 15 samples (Additional file 4: Figure S2).

### Weighted gene co-expression network construction

**Fig. 2:**
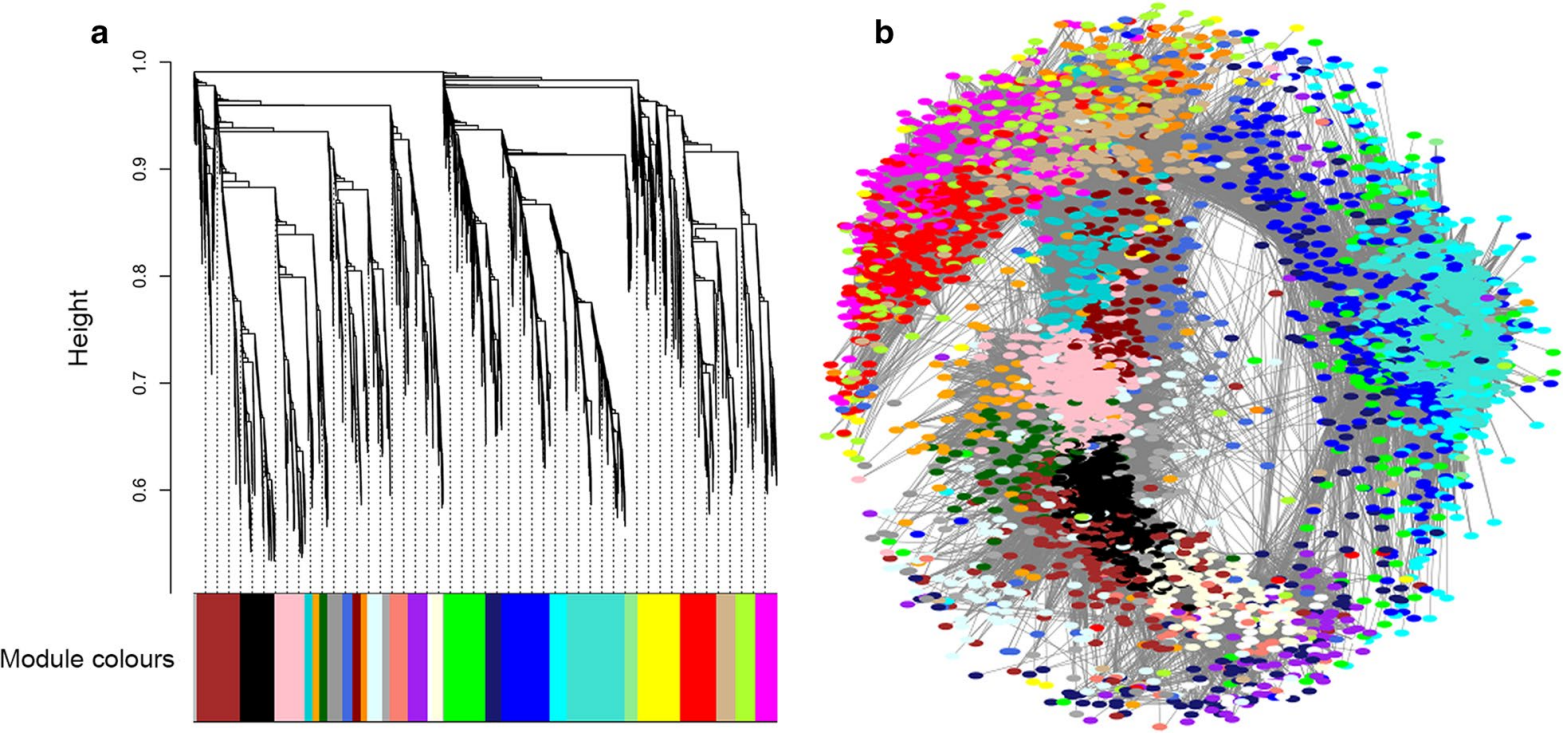
An illustration of the identified gene co-expression network modules in *T. brucei.* **a** Hierarchical cluster dendrogram. The *x*-axis represents the co-expression distance of the genes, while the *y*-axis represents the genes. A dynamic tree cutting algorithm identified the modules by splitting the tree at significant branching points. Modules are represented by different colors as shown by the dendrogram. **b** Co-expression network from weighted gene co-expression network analysis (WGCNA) based on topological overlap measures (TOMs) > 0.3 for visualization. Each point (or node) on the network represents a gene, and points of the same color form a gene module. Lines (edges) on the network connecting the nodes represent a relationship between the genes

**Fig. 3:**
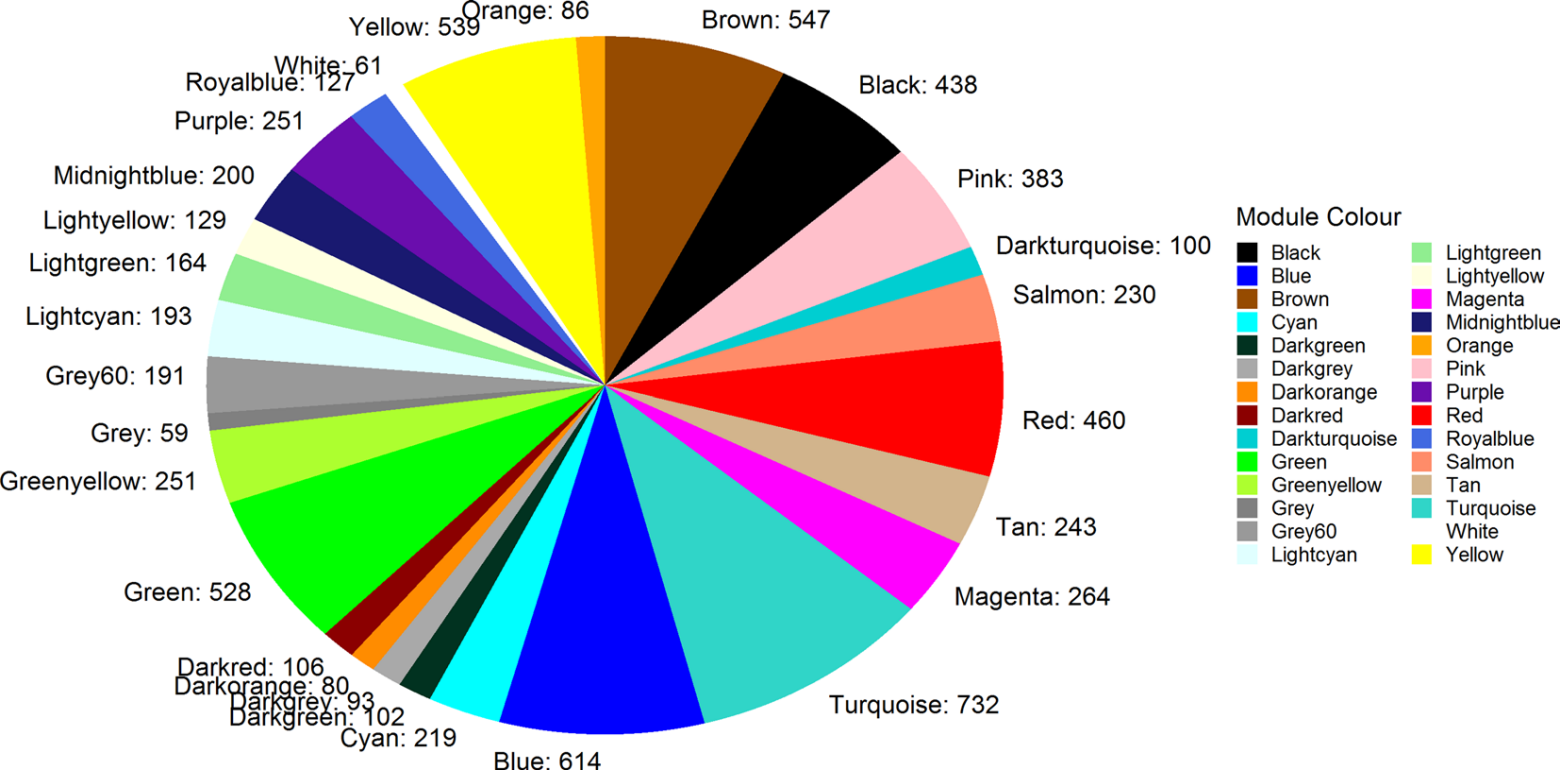
Number of genes identified in each module. In total, there were 28 modules. The gray module contains 59 genes that could not be assigned to any module and was excluded from downstream analysis

A total of 7390 protein coding genes from 15 samples were used for the construction of the co-expression network. Prior to generation of the network, the soft-thresholding power to which co-expression similarity was raised to calculate adjacency was determined by analysis of thresholding powers from 1 to 20. Power 14, the power for which the scale-free topology fitting index (R^2^) was ≥ 0.8, was chosen (Additional file 5: Figure S3). A total of 28 distinct modules were generated for 7390 protein coding genes from the hierarchical clustering tree (dendrogram) using the dynamic tree cut algorithm (Figs. 2, 3, and Additional file 6: Table S2). The gray module, which contained 59 genes that could not be assigned to any module, was excluded from the analysis (Fig. 3). Thus, a total of 27 modules were used in the subsequent analysis. The module with the least genes (61) was the white module while the turquoise module had the largest number of genes (732) (Fig. 3).

### Functional and pathway enrichment analysis

Out of the 27 modules generated, only 14 modules were found to be enriched for GO terms; 12 were over-represented and 2 (blue and green modules) were under-represented for GO terms (Additional file 7: Table S3). Seven of the 27 modules were enriched following KEGG pathway enrichment analysis, from which 5 were over-represented and 2 (lightcyan and blue modules) were under-represented for KEGG pathway terms (Additional file 8: Table S4). The top enriched GO terms for the modules with over-represented GO terms highlight some functions of the module genes (Table 1). Of the 12 modules with over-represented GO terms, 4 modules were over-represented for KEGG pathway terms and 1 module (yellow module) was over-represented for a KEGG pathway term (endocytosis), but not GO terms (Table 1).

**Table 1:** Modules with over-represented GO terms and their most significant over-represented GO and KEGG pathway terms

Module	Top enriched GO term	GO term adjusted *p* value	KEGG pathways term	KEGG term adjusted *p*-value
Brown	Adenylate cyclase activity	1.151E−02	_	_
Black	Cellular nitrogen compound biosynthetic process	2.779E−07	_	_
Pink	Cytochrome complex	4.917E−02	RNA transport	3.187E−02
Darkturquoise	Transferase activity, transferring phosphorus-containing groups	1.815E−02	_	_
Salmon	RNA binding	2.191E−03	_	_
Purple	Mitochondrial protein complex	1.093E−06	_	_
Lightyellow	Structural constituent of ribosome	6.262E−11	Ribosome	9.416E−08
Turquoise	Cell surface	2.395E−04	_	_
Red	Cytoskeletal part	7.976E−04	Homologous recombination	1.478E−02
Tan	Spindle pole	4.930E−02	_	_
Greenyellow	Cytoskeleton	1.211E−03	_	_
Magenta	Cytoskeleton	3.979E−04	Purine metabolism	9.042E−03
Yellow	_	_	Endocytosis	2.502E−02

“_” indicates detection of no significant GO or KEGG terms

### Modules hub gene identification

Highly connected genes in a module are referred to as intra-modular hub genes. These hub genes are considered functionally significant in the enriched functions of the modules. Following the hypothesis that higher connectivity for a gene implies more importance in the module’s functional role, genes with the highest connectivity in the 27 modules were determined and considered to be the hub genes (Additional file 9: Table S5). Hub genes for the 12 modules with over-represented GO terms are described in Table 2.

**Table 2:** Identified hub genes and their encoding proteins for the 12 modules with over-represented GO terms

Module	Hub gene	Encoding protein
Brown	Tb927.11.1570	Hypothetical protein, conserved
Black	Tb927.7.1790	Adenine phosphoribosyltransferase, putative
Pink	Tb927.10.6200	Hypothetical protein, conserved
Darkturquoise	Tb927.8.6650	RNA-binding protein, putative
Salmon	Tb927.11.1450	2-Oxoglutarate dehydrogenase E1 component, putative
Purple	Tb927.1.600	Phosphate-repressible phosphate permease, putative
Lightyellow	Tb927.10.2560	Mitochondrial malate dehydrogenase
Red	Tb927.7.6920	Hypothetical protein, conserved
Tan	Tb927.3.2930	RNA-binding protein RBP6, putative
Greenyellow	Tb927.7.920	Inner arm dynein 5-1
Magenta	Tb927.9.6290	Arginine kinase
Turquoise	Tb927.9.15630	BARP protein

### 3′ UTR motif prediction based on gene co-expression modules

**Fig. 4:**
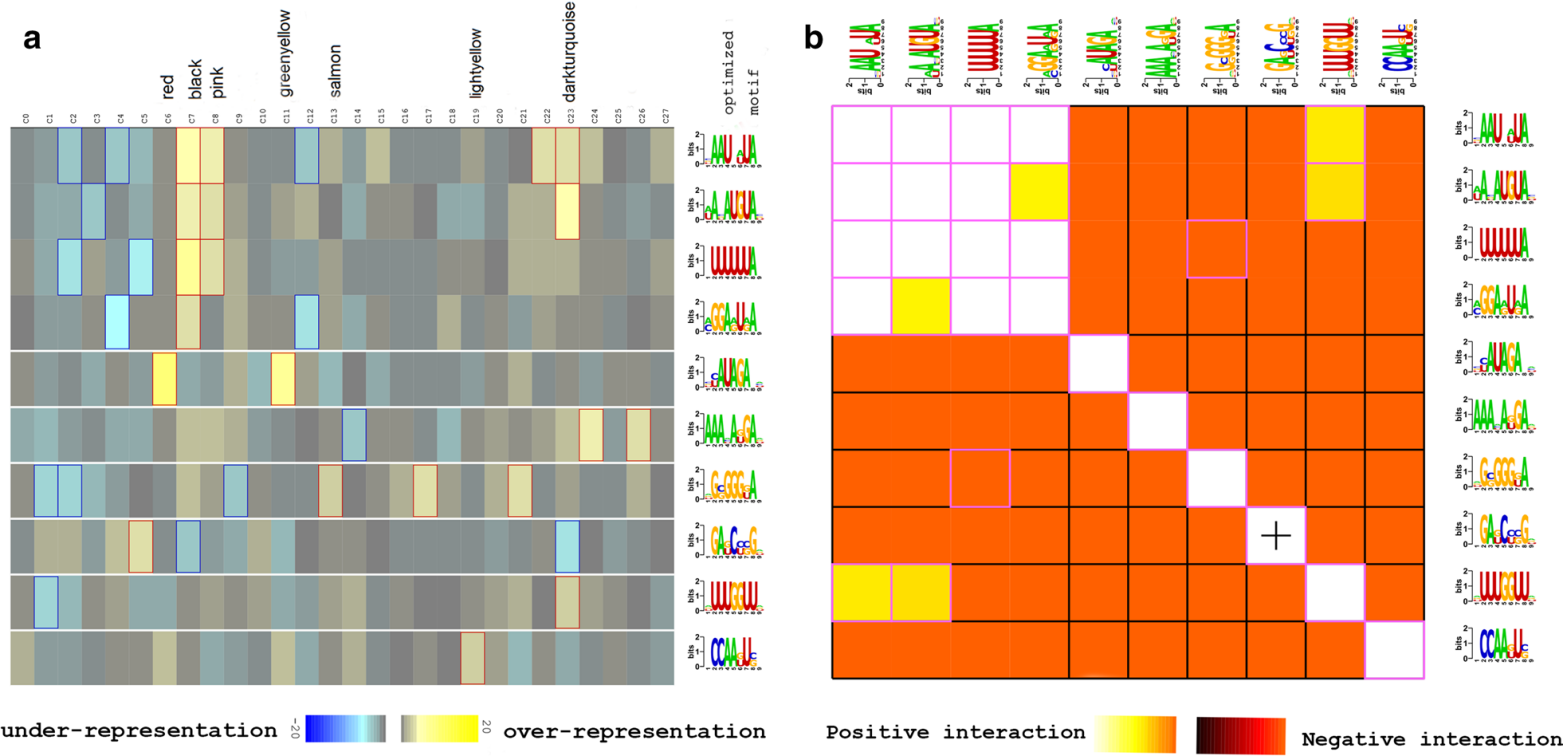
Prediction of regulatory elements in the 3′ untranslated regions (UTR) based on gene co-expression modules. **a** Predicted motifs for the gene modules are shown. Columns represent gene modules, while rows represent the predicted motifs with consensus sequence on the right side. Over-representation of a motif for a given gene module is indicated by yellow color with significant over-representation highlighted by red frames. Blue color map and frames indicate under-representation. **b** Motif pairs co-occurring in the 3′ UTR are shown in the heatmap where each row and each column correspond to a predicted motif. Light colors indicate the presence of another motif within the same 3′ UTR while dark colors indicate that the motifs are absent in the same 3′ UTR. “+” indicates significant spatial co-localization between pairs of motifs

Genes in a given module are hypothesized to be co-regulated as they are assumed to have similar functions. Consequently, their *cis*-regulatory element should be similar. Following this hypothesis, ten statistically significant RNA motifs, each over-represented in different gene modules, were identified using FIRE (Fig. 4a).

## Discussion

This study employed the WGCNA method [17] to construct the *T. brucei* weighted gene co-expression network using RNA-Seq data. The resulting co-expression network analysis allowed identification of modules (gene clusters) as well as enrichment analysis in GO [15] and KEGG [16] annotation databases to associate the modules with their functions. Highly connected genes in a module, known as intra-modular hub genes [17], were also determined as they are key drivers of a molecular process or act as a representative of the predominant biological function of the module. Here, we demonstrate the usefulness of the network for functional genomic analysis using an example of the cell cycle and protein biosynthesis enriched functions.

The cell cycle in eukaryotes comprises four phases, namely: G_0_/G_1_, S, G_2_, and M phases [36]. The cell prepares for division in the first gap phase (G_0_/G_1_), replicates the DNA during the S phase, and then undergoes mitosis (M) in the second gap phase (G_2_). In *T. brucei*, the cell cycle is tightly regulated to ensure that single-copy organelles and structures such as the Golgi body, mitochondrion, kinetoplast, nucleus, basal body, and flagellum are duplicated, maintained at precise positions in the cell and segregated accurately [37]. Various GO terms related to organelles were over-represented in the black module (Figs. 2 and 3) and included microbody, peroxisome, glycosome, and acidocalcisome (Additional file 10: Figure S4). The organelles duplicate in the first gap phase (G_0_/G_1_) [38]. This suggests that genes assigned to the black module (Figs. 2 and 3) could play a role in the cell cycle particularly during the G_0_/G_1_ phase. Furthermore, some cyclins and cdc2-related kinases (CRKs) that are key regulators of the cell cycle such as CYC2 (Tb927.11.14080), CYC5 (Tb927.10.11440), and CRK10 (Tb927.3.4670) were assigned to the black module (Additional file 6: Table S2). Both CY2 and CY5 regulate the transition of G_1_ phase to S phase [39]. Co-expression of CRK10, whose regulatory role is presently unknown, with CYC2 and CYC5 and its demonstrated interaction with CYC2 in yeast two-hybrid assay [39] suggests a possible role in G_1_ to S phase transition.

The hub gene for the black module was identified as adenine phosphoribosyltransferase (APRT) (Table 2), which plays a crucial role in the purine salvage pathway in *T. brucei*. This parasite lacks a de novo purine biosynthetic pathway [40]. Purine nucleotides are precursors of DNA and RNA and are also constituents of second messengers in signaling pathways such as cyclic AMP [41]. In this regard, APRT may be important in enriched module functions such as cyclic nucleotide biosynthesis and synthesis of the structural constituent of the ribosome, particularly ribosomal RNA, and consequently, signaling and protein biosynthesis. Signaling is depicted by the black module’s over-represented GO terms such as adenylate cyclase activity, while protein biosynthesis is depicted by GO terms such as translation, unfolded protein binding, protein folding, and structural constituent of ribosome (Additional file 10: Figure S4).

The red module (Figs. 2 and 3) was functionally enriched for GO terms such as DNA replication and chromosome organization and the KEGG pathway term homologous recombination, indicating that its genes are involved in the progression of the cell cycle (Additional file 11: Figure S5 and Table 1). Additionally, the red module has some genes involved in cytokinesis such as BOH1 (Tb927.10.12720), which cooperates with *Tb*PLK to initiate cytokinesis and flagellum inheritance [42], and cytokinesis initiation factor 2 (CIF2) (Tb927.9.14290), which is involved in initiation of cytokinesis [43] (Additional file 6: Table S2). Other genes assigned to this module were in concordance with the enriched functions. These were nucleus- and spindle-associated protein 1 (NuSAP1) (Tb927.11.8370), which is required in chromosome segregation and NuSAP2 (Tb927.9.6110), which promotes the G_2_/M transition [44]. The hub gene for the red module is a hypothetical gene (Tb927.7.6920), which may play a key role in the progression of the cell cycle.

The tan module (Figs. 2 and 3), whose enriched GO terms include the spindle pole and microtubule cytoskeleton, has genes such as CIF4 (Tb927.10.8240), TLK1 (Tb927.4.5180), and FPRC (Tb927.10.6360), which are involved in cytokinesis [45, 46]. The hub gene for the tan module is RNA-binding protein RBP6 (Table 2). Interestingly, over-expression of RBP6 *in vitro* has been demonstrated to recapitulate the parasite’s tsetse fly stage developmental form, which was previously elusive in culture [47]. However, the exact role of RBP6 during the parasite’s development in the tsetse fly is yet to be elucidated. Based on its assignment to the tan module, it is likely to be involved in regulating a key step during progression of the cell cycle.

The salmon module (Figs. 2 and 3) has enriched functions in RNA metabolic processing depicted by the module’s enriched GO terms, which are RNA metabolism, nucleic acid binding, and RNA binding (Additional file 12: Figure S6). RNA binding may either involve binding of the mRNA by RNA-binding proteins (RBPs) as a post-transcriptional gene regulation mechanism in *T. brucei* [48, 49] or binding by translation initiation factors for protein synthesis [50]. The salmon module has translation initiation factor eIF4E1 (Tb927.11.2260) and poly(A) binding protein PABP2 (Tb927.9.10770), which have previously been shown to be co-localized in *T. brucei* [51]. An RNA-binding protein related to the stress response, ZC3H30 (Tb927.10.1540), together with an associated stress response granule (Tb927.8.3820) [52], was assigned to the salmon module. The hub gene for the salmon module is the 2-oxoglutarate dehydrogenase E1 component (Table 2). 2-Oxoglutarate dehydrogenase is an enzyme involved in the tricarboxylic acid (TCA) cycle in the mitochondrion implicated in the degradation of proline and glutamate to succinate, which can enter the gluconeogenesis pathway in procyclic trypanosomes [53]. This hub gene could be important in the role of the mitochondrion in responding to stress as a result of change in energy source in insect-stage trypanosomes.

Some genes that were identified as hub genes had previously been characterized through functional studies. These include: inner arm dynein 5-1 (IAD5-1) (Tb927.7.920), which was identified as a hub gene for the greenyellow module. Knockdown of IAD5-1 through RNAi was shown to cause a defect in cell motility, indicating its functional role in the parasite’s motility [54]. Yet another inner arm dynein gene, DNAH10 (Tb927.4.870), has been implicated in cell motility through RNAi knockdown functional studies [55]. In our study, both DNAH10 and IAD5-1 clustered in the greenyellow module which was highly enriched for GO terms associated with cytoskeleton and motility (Additional file 13: Figure S7), confirming previous functional roles in cell motility by [54, 55].

Additionally, RNAi knockdown of DRBD13 (Tb927.8.6650) has previously been found to be deleterious to parasite’s growth in the tsetse fly by causing up-regulation of RBP6 (Tb927.3.2930) gene [56]. Both DRBD13 and RBP6 were identified as hub genes in the constructed network: DRBD13 was found in the darkturquoise module enriched for transfer of phosphorus-containing groups and RBP6 in the tan module enriched for cell cycle terms such as spindle pole. The RBP6 has been implicated in parasite development in the tsetse fly [47]. Its identification as a hub gene and congruence in its predicted function through this study confirms the usefulness of such *in silico* studies in selecting candidates for *in vitro* studies.

Regulation of gene expression in *T. brucei* occurs almost exclusively post-transcriptionally as a result of polycistronic arrangement of their genes [50, 57, 58]. Post-transcriptional regulation of mRNA abundance mainly involves interaction of their *cis*-regulatory element and a *trans*-acting element such as an RNA-binding protein [59]. Genes with similar functions are co-regulated together; thus, their mRNAs are hypothesized to have similar *cis*-regulatory elements [19]. Since the gene modules of a co-expression network are composed of genes with similar functions, they can be used as a basis for identifying potential regulatory elements in the untranslated regions of mRNA.

Two motifs ([AU]A[CGU]AUGUA[CGU] and [CGU][CU]AUAGA.[ACU]) that had consensus sequences similar to previously identified motifs were found to be over-represented (Fig. 4a). The motif [AU]A[CGU]AUGUA[CGU] contains the core sequence, UGUA, that is recognized by the PUF family of RNA-binding proteins [60] and has previously been identified in *T. brucei* as targeting transcripts involved in the cell cycle [61–63]. The motif was over-represented in the black, pink, and darkturquoise modules (Fig. 4a). [AU]A[CGU]AUGUA[CGU] co-occurs with other motifs including [CGU]AAU.[AU]UA.,.UUUUUUA., [AC]GGA[AG]U[AG]A. and [AGU]UUUGGUU[AGU] (lighter colors in Fig. 4b). Co-occurrence of motifs means that they co-localize within the same untranslated region (UTR), which indicates that the presence of one motif implies the presence of its putative counterpart [19]. These co-occurring motifs may provide further information on post-transcriptional regulation. For instance, co-localization of two motifs close to each other on a transcript could imply physical interaction of their binding elements, hence their functional interaction [19].

The other motif, [CGU][CU]AUAGA.[ACU], was over-represented in the red and greenyellow modules (Fig. 4a). This consensus motif contains the core AUAGA sequence similar to CAUAGAA that has been implicated in cell cycle regulation [64, 65] and was previously predicted in *T. brucei* [63]. Notably, genes in the red module were enriched for cell cycle functions while those in the greenyellow module were enriched for microtubule-associated functions, including motility (Additional file 13: Figure S7). Motility in *T. brucei* is mediated through the flagellum [66]. Importantly, flagellum motility is essential for completion of the cell division [67, 68] suggesting co-regulation of genes in the greenyellow module together with those in the red module. The motif [CGU][CU]AUAGA.[ACU] does not co-occur with other motifs, which possibly suggests that its functions have opposing effects compared with functions of the other motifs (Fig. 4b). Overall, characterization of these identified *cis*-regulatory elements will advance our knowledge on post-transcription gene regulation and provide potential chemotherapeutic targets against key regulatory functions in *T. brucei* for disease control.

## Conclusions

Construction of the *T. brucei* gene co-expression network provides a valuable resource for identifying candidate genes for experimental work. These candidate genes could be important in elucidating molecular mechanisms that underlie important biological processes during the parasite’s development in tsetse fly. Our results indicate correspondence between the enriched functions of module genes, particularly the identified hub genes, and known *T. brucei* biology. This illustrates the effectiveness of the co-expression network analysis as an approach to explore functionally relevant genes in *T. brucei* development in tsetse fly. Furthermore, the hub genes from this study that encode proteins whose functional roles are still unknown could be used to inform research focus and priorities while performing *in vitro* studies. Knowledge on *T. brucei* development in the tsetse fly vector is crucial in identifying key targets to block transmission of these medically and economically important parasites.

## Supplementary information

**Additional file 1.** Code used in data pre-processing, network construction, and functional analysis. It is archived at https://github.com/wanjauk/tbrucei_gcn.

**Additional file 2: Table S1**. Sample metadata for samples used in this study. Samples highlighted in red were excluded from analysis because of failing quality assessment

**Additional file 3: Figure S1**. Principal component analysis (PCA) and Pearson correlation heatmap prior to accounting for batch effects. (**a**) Each point in the PCA plot represents an experimental sample, and point color indicates a batch that consists of the biological replicates. (**b**) Color codes along the left side of the sample correlation heatmap indicate samples based on the batch they belong to. MG1 and MG2 are midgut samples, PV2 are proventriculus samples, and SA2 are salivary gland samples

**Additional file 4: Figure S2**. Assessment of per-gene read count distribution per sample using a boxplot. The distribution of per-gene read counts per sample showed a median steady-state expression level of ~ 6.5 log_2_ counts per million for all the 15 samples

**Additional file 5: Figure S3.** Scale-free topology plot for selecting the power β for the signed correlation network. (**a**) Scale free topology index (*y* axis) as a function of powers, β, 1 to 20 (*x* axis). (**b**) Mean connectivity (*y* axis) as a decreasing function of powers β (*x* axis)

**Additional file 6: Table S2.** Co-expression network genes comprising the modules of the network. There are a total of 7390 genes distributed across 28 modules in the co-expression network

**Additional file 7: Table S3.** Module GO enrichment results; 14 modules with their significantly over- and under-represented GO terms are tabulated

**Additional file 8: Table S4.** Module KEGG enrichment results; 7 modules with their significantly over- and under-represented KEGG terms are tabulated

**Additional file 9: Table S5.** Hub genes for all the 27 modules and the proteins they encode

**Additional file 10: Figure S4.** Black module over-represented GO terms. (**a**) Biological process GO terms; (**b**) cellular component GO terms; (**c**) molecular function GO terms

**Additional file 11: Figure S5**. Red module over-represented GO terms. (**a**) Biological process GO terms; (**b**) cellular component GO terms

**Additional file 12: Figure S6**. Salmon module over-represented GO terms. (**a**) Biological process GO terms; (**b**) molecular function GO terms

**Additional file 13: Figure S7.** Greenyellow module over-represented GO terms. (**a**) Biological process GO terms; (**b**) cellular component GO terms; (**c**) molecular function GO terms

## Data Availability

The datasets supporting the conclusions of this study are included within the article and in the additional data files.

## Abbreviations

AK1: Arginine kinase 1
APRT: Adenine phosphoribosyltransferase
BARP: Brucei alanine-rich protein
BOH1: Bait on hook 1
CIF: Cytokinesis initiation factor
CRK: cdc2-related kinase
ENA: European Nucleotide Archive
FIRE: Finding informative regulatory elements
GO: Gene Ontology
KEGG: Kyoto Encyclopedia of Genes and Genomes
PABP: Poly(A) binding protein
PCA: Principal component analysis
RBP6: RNA-binding protein 6
TMM: Trimmed mean of M-values
TOM: Topological overlap matrix
UTR: Untranslated region
WGCNA: Weighted gene correlation network analysis
