# Supplementary material for: Gene co-expression network analysis of Trypanosoma brucei in tsetse fly vector
Source: Parasit Vectors. 2021 Jan 22;14:74. doi: 10.1186/s13071-021-04597-6 (PMC7821691; doi:10.1186/s13071-021-04597-6)
Supplement: Supplementary file 1 — Additional file 1. Code used in data pre-processing, network construction, and functional analysis. It is archived at https://github.com/wanjauk/tbrucei_gcn. [file 13071_2021_4597_MOESM1_ESM.pdf]

Trypanosoma brucei co-expression network analysis

Kennedy Mwangi

September 13, 2020

Introduction

This document contains the workflow used in the analysis of *T. brucei* gene co-expression network analysis. It contains code used in each step of the analysis. The code and output files are archived at https://github.com/wanjauk/tbrucei_gcn

Setting up R for the analysis

```r
# load required R packages -------------------------------------------------

library("here")
library("tidyr")
library("dupRadar")
library("Rsubread")
library("limma")
library("edgeR")
library("sva")
library("RColorBrewer")
library("ggplot2")
library("gplots")
library("reshape2")
library("ggfortify")
library("openxlsx")
library("WGCNA")
library("flashClust")
library("tidyverse")
library("igraph")
library('foreach')
library('doParallel')
library('goseq')
library('Trypanosoma.brucei.TREU927', character.only = TRUE)
library('org.Tb927.tritryp.db')
library('TxDb.TbruceiTREU927.tritryp43.genes')
library('dplyr')
library('rtracklayer')
library('tibble')
library('ggrepel')
library("knitr")
library("readxl")
```

```r
# ensure results are reproducible
set.seed(1)

# other settings
options(digits = 4)
options(stringsAsFactors = FALSE)
```

```r
# set number of threads to allow in WGCNA analysis
allowWGCNAThreads(nThreads=20)
```

Data acquisition

Data used in this study is obtained from European Nucleotide Archive under accession number SRP002243 and SRR965341.

First, metadata for the data is obtained from EBI as follows:

```r
#Obtain FASTQ urls to download the data used in this study from ENA database.

# ENA metadata
# code adopted from:
# https://wiki.bits.vib.be/index.php/Download_read_information_and_FASTQ_data_from_the_SRA

# accession numbers from Savage et al and Telleria et al the studies
accessions <- c("SRP002243","SRR965341")

# create directories to store the files if they don't exist.
telleria_dir <- here::here("data", "raw", "telleria")
if (!dir.exists(telleria_dir)) {
  dir.create(telleria_dir, recursive=TRUE)
}

savage_dir <- here::here("data", "raw", "savage")
if (!dir.exists(savage_dir)) {
  dir.create(savage_dir, recursive=TRUE)
}

for (accession_num in accessions) {

  # these samples from Savage et al are single-end reads.
  if (accession_num == "SRP002243"){

    # construct the url to ENA database
    ena.url <- paste("https://www.ebi.ac.uk/ena/portal/api/filereport?accession=",
                     accession_num,
                     "&result=read_run",
                     "&fields=study_accession,sample_accession,",
                     "experiment_accession,run_accession,scientific_name,fastq_ftp,",
                     "submitted_ftp,sra_ftp",
                     "&download=true",
                     sep="")

    # Load the metadata from ENA
    ENA.metadata <- read.table(url(ena.url), header=TRUE, sep="\t")

    # get the fastq urls
    fastq.urls <- ENA.metadata[grepl("fastq_ftp", names(ENA.metadata))]

    # create a text file with urls to fastq files in ENA database
    write.table(fastq.urls, here::here(savage_dir, "savage.fastq.urls.txt"),
```

```
                eol = "\n",
                quote = FALSE,
                col.names = FALSE,
                row.names = FALSE)

  } else {

    # The other sample (SRR965341) from Telleria et al study has paired-end reads.
    #
    # construct the url to ENA database
    ena.url <- paste("https://www.ebi.ac.uk/ena/portal/api/filereport?accession=",
                     accession_num,
                     "&result=read_run",
                     "&fields=study_accession,sample_accession,",
                     "experiment_accession,run_accession,scientific_name,fastq_ftp,",
                     "submitted_ftp,sra_ftp",
                     "&download=true",
                     sep="")

    # Load the metadata from ENA
    ENA.metadata <- read.table(url(ena.url), header=TRUE, sep="\t")

    # ensure that R1 and R2 fastq files are in separate rows
    ENA.metadata <- ENA.metadata %>% separate_rows(fastq_ftp, sep=";")

    # get the fastq urls
    fastq.urls <- ENA.metadata[grepl("fastq_ftp", names(ENA.metadata))]

    # create a text file with urls to fastq files in ENA database
    write.table(fastq.urls, here::here(telleria_dir, "telleria.fastq.urls.txt"),
                eol = "\n",
                quote = FALSE,
                col.names = FALSE,
                row.names = FALSE)
  }

}
```

Prepare the samples metadata.

```
# Load SRA metadata from Savage et al 2016 study
SRA.metadata <- read.table(here::here("data", "raw", "savage","SraRunTable.metadata.txt"),
                           header = TRUE, sep = "\t")

# obtain sample metadata to be used later in analysis in R.
matches <- c("Run","Library_Name","Sample_Name")
samples.metadata <- SRA.metadata[grepl(paste(matches, collapse="|"), names(SRA.metadata))]

# create a grouping factor that will place each sample in the one of three tissues i.e.
# midgut (MG), proventriculus(PV) and salivary glands (SG)
tissue <- factor(c("MG", "MG", "MG", "MG", "MG", "PV", "PV", "SG", "SG", "SG", "SG",
                   "MG", "MG", "PV", "SG", "SG", "PV"))

# append factor to samples.metadata to group samples
```

```r
samples.metadata["Tissue"] <- tissue

# Add sample from Telleria et al 2014 study (SRR965341) to sample metadata.
samples.metadata$Run <- as.character(samples.metadata$Run)
samples.metadata <- rbind(samples.metadata, "18" = c("SA2", "SRR965341", "SA2", "SG"))

# include batch information for the 18 samples
batch <- factor(c(1, 1, 1, 2, 2, 2, 2, 1, 1, 2, 2, 1, 2, 2, 1, 2, 2, 2))
samples.metadata["Batch"] <- batch

# save the metadata to an R object
saveRDS(samples.metadata, here::here("data", "raw", "samples.metadata.RDS"))
```

```r
# print out the sample metadata table
samples.metadata
```

```
##    Library_Name       Run Sample_Name Tissue Batch
## 1           mg1 SRR039378         MG1     MG     1
## 2           mg1 SRR039381         MG1     MG     1
## 3           mg1 SRR039453         MG1     MG     1
## 4        MG2_SL SRR039454         MG2     MG     2
## 5        MG2_SL SRR039455         MG2     MG     2
## 6        PV2_SL SRR039456         PV2     PV     2
## 7        PV2_SL SRR039457         PV2     PV     2
## 8           SA1 SRR039937         SA1     SG     1
## 9           SA1 SRR039938         SA1     SG     1
## 10       SA2_SL SRR039939         SA2     SG     2
## 11       SA2_SL SRR039940         SA2     SG     2
## 12          mg1 SRR039948         MG1     MG     1
## 13       MG2_SL SRR039949         MG2     MG     2
## 14       PV2_SL SRR039950         PV2     PV     2
## 15          SA1 SRR039951         SA1     SG     1
## 16       SA2_SL SRR039952         SA2     SG     2
## 17       PV2_SL SRR042429         PV2     PV     2
## 18          SA2 SRR965341         SA2     SG     2
```

Next, RNASeq data is downloaded from EBI database's FTP site. Some of the downstream tools require that FASTQ files that were downloaded in zipped form are unzipped.

```bash
#!/bin/bash
#
#Script to download fastq files from European Nucleotide Archive
#
#USAGE:
# ./download-fastq-files.sh \
# ../../data/raw/savage/savage.fastq.urls.txt /data/kwanjau/savage ;
# ./download-fastq-files.sh \
# ../../data/raw/telleria/telleria.fastq.urls.txt /data/kwanjau/telleria
#
FILE=$1 #File containing fastq url links to EBI FTP site

OUT_DIR=$2

cat ${FILE} | xargs -n1 wget $3 -P ${OUT_DIR}
```

```
#decompress fastq.gz files
#
FASTQ_FILES=${OUT_DIR}/*.fastq.gz

for file in ${FASTQ_FILES}; do
    gunzip ${file}
done
```

Downloading *T. brucei* and *G. morsitans* genome and annotation files

Genomes are obtained from their respective databases before alignment. The genome and annotation files are downloaded from the TriTrypDB and vectorbase databases as follows:

```
#Downloading T. brucei genome

wget https://tritrypdb.org/common/downloads/release-43/TbruceiTREU927/fasta/data/TriTrypDB-43_TbruceiTR
-P ../../data/scratch/tbrucei/

#Downloading the GFF file
wget https://tritrypdb.org/common/downloads/release-43/TbruceiTREU927/gff/data/TriTrypDB-43_TbruceiTREU
-P ../../data/scratch/tbrucei/

# convert the tbrucei gene annotation from GFF format to GTF (required by some downstream tools)
# uses gffread from cufflinks
gffread ../../data/scratch/tbrucei/TriTrypDB-43_TbruceiTREU927.gff \
-T -o ../../data/scratch/tbrucei/TriTrypDB-43_TbruceiTREU927.gtf

# Download T. brucei annotated transcripts (for use in UTR motif discovery)
wget https://tritrypdb.org/common/downloads/release-43/TbruceiTREU927/fasta/data/TriTrypDB-43_TbruceiTR
-P ../../data/scratch/tbrucei/

# Downloading Glossina genome --Moved to new loaction after VEuPathDB creation
# wget https://www.vectorbase.org/download/glossina-morsitans-yalescaffoldsgmory1fagz \
# -P ../../data/scratch/glossina/

# Downloading GTF file --Moved to new loaction after VEuPathDB creation
# wget https://www.vectorbase.org/download/glossina-morsitans-yalebasefeaturesgmory19gtfgz \
# -P ../../data/scratch/glossina/

# Downloading Glossina genome
wget https://vectorbase.org/common/downloads/Pre-VEuPathDB%20VectorBase%20files/Glossina-morsitans-Yale
-P ../../data/scratch/glossina/

# Downloading GTF file
wget https://vectorbase.org/common/downloads/Pre-VEuPathDB%20VectorBase%20files/Glossina-morsitans-Yale
-P ../../data/scratch/glossina/

# unzip Glossina genome file
gunzip ../../data/scratch/glossina/Glossina-morsitans-Yale_SCAFFOLDS_GmorY1.fa.gz

# unzip Glossina annotation file file
gunzip ../../data/scratch/glossina/Glossina-morsitans-Yale_BASEFEATURES_GmorY1.9.gtf.gz
```

Data quality assessment

After downloading the RNASeq data, its quality is checked through the FASTQC tool whose output is a report in HTML format.

```
# #!/bin/bash
#
#Script to generate FastQC reports using the FastQC tool
#
# USAGE:
# bash generate-fastqc-reports.sh ../../results/figures/fastqc_reports/savage ../../data/raw/savage
# bash generate-fastqc-reports.sh ../../results/figures/fastqc_reports/telleria ../../data/raw/telleria
#
# make directory to store the results
mkdir -p ../../results/figures/fastqc_reports/savage/;
mkdir -p ../../results/figures/fastqc_reports/telleria/
#
#load fastqc module
# module load fastqc/0.11.4

# fastqc reports directory
REPORT_DIR=$1

# fastq files directory
FASTQ_DIR=$2

for file in $FASTQ_DIR/*.fastq; do
   fastqc ${file} -o ${REPORT_DIR} -f fastq
done
```

Following the high rate of duplicate reads after FASTQC analysis, further analysis is done to ascertain their cause. Duplicate reads are assessed whether they arise from artifacts in PCR (PCR duplicates) or from biological causes (highly expressed genes). This is done later in the analysis after read mapping.

Concatenate and index the *T. brucei* and *G. morsitans* genome files

T. brucei and *G. morsitans* genome files are concatenated into a single fasta file which is used during the alignment of the reads. This ensures no cross-mapping of reads take place during alignment with HISAT2. The next step is indexing the genome using HISAT2.

```
# #!/bin/bash

# USAGE:
# ./concatenate-and-index-genome.sh \
# ../../data/scratch/concatenated_genomes/brucei-morsitans_genomes.fasta \
# ../../data/scratch/indexed_genome

# make a directory to store the concatenated and indexed genomes
 mkdir -p ../../data/scratch/concatenated_genomes
 mkdir -p ../../data/scratch/indexed_genome

# concatenate the genome files
cat ../../data/scratch/tbrucei/TriTrypDB-43_TbruceiTREU927_Genome.fasta \
../../data/scratch/glossina/Glossina-morsitans-Yale_SCAFFOLDS_GmorY1.fa \
> ../../data/scratch/concatenated_genomes/brucei-morsitans_genomes.fasta
```

```
# concatenate the gtf annotation files
cat ../../data/scratch/tbrucei/TriTrypDB-43_TbruceiTREU927.gtf \
../../data/scratch/glossina/Glossina-morsitans-Yale_BASEFEATURES_GmorY1.9.gtf \
> ../../data/scratch/concatenated_genomes/brucei-morsitans_annotations.gtf

#
#index genome using HISAT2
#
GENOME_FILE=$1

INDEX_DIR=$2

hisat2-build ${GENOME_FILE} ${INDEX_DIR}/bru-mor_genome_index_hisat2
```

Aligning the reads to the genome (Read Mapping)

Alignment of the reads to the chimeric genome. The output is SAM files.

```
# #!/bin/bash
#
#Script to align reads to the indexed genome using HISAT2
#

#USAGE:
# ./reads-alignment.sh \
# ../../data/raw/savage \
# ../../data/scratch/indexed_genome \
# ../../data/scratch/reads-alignment-output/savage \
# ../../data/raw/telleria/SRR965341_1.fastq \
# ../../data/raw/telleria/SRR965341_2.fastq \
# ../../data/scratch/reads-alignment-output/telleria

# create alignment output directory
mkdir -p ../../data/scratch/reads-alignment-output/savage
mkdir -p ../../data/scratch/reads-alignment-output/telleria

# Fastq directory
FASTQ_DIR=$1

# genome index directory
INDEX_DIR=$2

# alignment output directory
ALIGN_OUT=$3

# telleria paired-end reads
READ1=$4
READ2=$5
TELLERIA_OUT=$6

for fastq in ${FASTQ_DIR}/*.fastq; do
    fqname=$(basename "$fastq" .fastq)
```

```
        hisat2 \
         -x ${INDEX_DIR}/bru-mor_genome_index_hisat2 \
         -U ${fastq} \
         -S ${ALIGN_OUT}/${fqname}.sam \
         -p 6 \
        --summary-file ${ALIGN_OUT}/${fqname}.txt \
        --new-summary
done


# Process paired-end data (Telleria's study)
telleria_fastq_name=$(basename "$READ1" _1.fastq)

hisat2 \
         -x ${INDEX_DIR}/bru-mor_genome_index_hisat2 \
         -1 ${READ1} \
         -2 ${READ2} \
         -S ${TELLERIA_OUT}/${telleria_fastq_name}.sam \
         -p 6 \
        --summary-file ${TELLERIA_OUT}/${telleria_fastq_name}.txt \
        --new-summary
```

Assessment of the duplication rate

At this point, quality control to assess the duplication rate can be performed. First, the SAM files are converted to sorted BAM files required by dupRadar tool. The BAM files are then sorted using samtools

```
# #!/bin/bash
#
#Script to convert sam files to sorted bam files
#
# USAGE:
# ./convert-sam-to-bam.sh \
# ../../data/scratch/reads-alignment-output/savage \
# ../../data/scratch/sam-to-bam-output/savage \
# ../../data/scratch/reads-alignment-output/telleria/SRR965341.sam \
# ../../data/scratch/sam-to-bam-output/telleria

# make directory for sorted bam files output
mkdir -p ../../data/scratch/sam-to-bam-output/savage
mkdir -p ../../data/scratch/sam-to-bam-output/telleria

# sam files directory
SAM_DIR=$1

# bam files directory
BAM_DIR=$2

# telleria files and output directory
SAM_FILE=$3
OUT_DIR=$4
```

```
# convert sam file to sorted bam files
for sam_file in ${SAM_DIR}/*.sam; do
    sam_file_name=$(basename "$sam_file" .sam)
        samtools view -S -b $sam_file | \
        samtools sort -o ${BAM_DIR}/${sam_file_name}.sorted.bam
done

# telleria paired-end data processing
# file name
tel_sam_file_name=$(basename "$SAM_FILE" .sam)

samtools view -S -b $SAM_FILE | \
samtools sort -o ${OUT_DIR}/${tel_sam_file_name}.sorted.bam
```

Next, duplicates are marked in the BAM files using Picard.

```
# #!/bin/bash
#
#Script to mark duplicates in BAM files using picard
#
# USAGE:
# ./mark-duplicates.sh \
# ../../data/scratch/sam-to-bam-output/savage \
# ../../data/scratch/mark-duplicates-output/savage

# ./mark-duplicates.sh \
# ../../data/scratch/sam-to-bam-output/telleria \
# ../../data/scratch/mark-duplicates-output/telleria

# crate a directory for mark duplicates output
mkdir -p ../../data/scratch/mark-duplicates-output/savage
mkdir -p ../../data/scratch/mark-duplicates-output/telleria

# sorted bam files directory
SORTED_BAM_DIR=$1

# mark duplicates output
MARK_DUPES_OUT=$2

for bam_file in ${SORTED_BAM_DIR}/*.sorted.bam; do
    bam_file_name=$(basename "$bam_file" .sorted.bam)

        picard MarkDuplicates \
            I=$bam_file \
            O=${MARK_DUPES_OUT}/${bam_file_name}.dupMarked.bam \
            M=${MARK_DUPES_OUT}/${bam_file_name}.dupMetrics.txt
done
```

Assessing the strandedness of the reads

Before this quality control can be performed, we need to verify whether the reads are stranded or not as this is a required parameter for dupRadar as well as HTSeq tool later in the analysis. This can be done using RSeQC package - An RNA-seq Quality Control Package. `infer_experiment.py` module is used in this case.

RSeQC documentation and tutorial can be found here.

First we convert *T. brucei* genome annotation GTF file into `bed` format required by RSeQC package. Then we use `infer_experiment.py` to verify strandedness using a few samples.

```
# convert GTF genome annotation to BED format using a custom script from:
#https://github.com/ExpressionAnalysis/ea-utils/tree/master/clipper/gtf2bed

# USAGE:
# # savage data
# ./check-reads-strandedness.sh \
# ../../data/scratch/tbrucei/TriTrypDB-43_TbruceiTREU927.gtf \
# ../../data/scratch/tbrucei/TriTrypDB-43_TbruceiTREU927.bed \
# ../../data/scratch/sam-to-bam-output/savage/SRR039381.sorted.bam

# # telleria data
# ./check-reads-strandedness.sh \
# ../../data/scratch/tbrucei/TriTrypDB-43_TbruceiTREU927.gtf \
# ../../data/scratch/tbrucei/TriTrypDB-43_TbruceiTREU927.bed \
# ../../data/scratch/sam-to-bam-output/telleria/SRR965341.sorted.bam

# GTF file
GTF_FILE=$1

# BED file directory
BED_FILE=$2

# BAM file
BAM_FILE=$3


# create a BED file from GTF
# ../utils/gtf2bed.pl $GTF_FILE > $BED_FILE

infer_experiment.py -i $BAM_FILE -r $BED_FILE

# output for savage's SRR039381.sorted.bam
# This is SingleEnd Data
# Fraction of reads failed to determine: 0.0000
# Fraction of reads explained by "++,--": 0.4058
# Fraction of reads explained by "+-,-+": 0.5942

# output for telleria's SRR965341.sorted.bam
# This is PairEnd Data
# Fraction of reads failed to determine: 0.0019
# Fraction of reads explained by "1++,1--,2+-,2-+": 0.4999
# Fraction of reads explained by "1+-,1-+,2++,2--": 0.4982
```

The next step is to run the dupRadar quality control analysis setting the `stranded` parameter as `FALSE` as the reads are not strand specific. Tutorial for the tool can be found here

For each of the 18 samples, dupRadar tool is used to perform quality control in R. Samples that had technical duplicates were excluded from sample metadata and further analysis.

```r
samples.metadata <- readRDS(file = here::here("data","raw","samples.metadata.RDS"))
source(here::here("scripts","analysis","libraries.R"))
```

```r
source(here::here("scripts","analysis","settings.R"))

# Parameters
gtf_file <- here::here("data","scratch","concatenated_genomes",
                       "brucei-morsitans_annotations.gtf")
stranded <- 0
paired <- FALSE # for savage's data
threads <- 10

# path to bam files data
bam_file <- list.files(here::here("data","scratch","mark-duplicates-output","savage"),
                       pattern = ".bam",
                       full.names = TRUE)

for (file in bam_file){

  #get the file name
  file_name <- gsub(pattern = "\\.dupMarked.bam$", "", basename(file))

  # Duplication rate analysis
  dm <- analyzeDuprates(file, gtf_file, stranded, paired, threads)

  #Plots
  png(filename = here::here("results","figures","duplication_rate",paste0(file_name,".png")))
  duprateExpDensPlot(DupMat=dm)
  title(paste0(file_name))
  dev.off()

  # # Boxplot
  # duprateExpBoxplot(DupMat=dm)

}

#########################################
# Telleria paired-end data processing
#########################################

#Parameters
telleria_paired <- TRUE

# path to bam files data
telleria_bam_file <- list.files(here::here("data","scratch","mark-duplicates-output","telleria"),
                       pattern = ".bam",
                       full.names = TRUE)
#get the file name
telleria_file_name <- gsub(pattern = "\\.dupMarked.bam$", "", basename(telleria_bam_file))

# Duplication rate analysis
dm <- analyzeDuprates(telleria_bam_file, gtf_file, stranded, telleria_paired, threads)

#Plots
png(filename = here::here("results","figures","duplication_rate",paste0(telleria_file_name,".png")))
duprateExpDensPlot(DupMat=dm)
```

```r
title(paste0(telleria_file_name))
dev.off()

# Exclusion of the following samples was done after analysis showed they
# failed quality control.

# remove 3 samples that have technical duplicates (SRR039951, SRR039937, SRR039938)
samples.metadata <- samples.metadata[-15,]
samples.metadata <- samples.metadata[-9,]
samples.metadata <- samples.metadata[-8,]

# save the samples metadata
saveRDS(samples.metadata, here::here("data","raw","samples.metadata.clean.RDS"))
```

Reads quantification

HTSeq tool is used to count reads that aligned to the *T. brucei* genome. *T. brucei* annotation file is used and therefore HTSeq excludes counting *G. morsitans* reads that aligned to *Glossina* genome. The output is a text file for each sample that contains the number of reads that were counted for each gene.

```bash
# #!/bin/bash
#
#Script to counts the number of reads aligned to T. brucei genome using HTSeq.
#Resource: HTSeq documentation https://htseq.readthedocs.io/en/latest/count.html
#
# module load htseq/0.11.2

# USAGE:
# ./reads-quantification.sh \
# ../../data/scratch/sam-to-bam-output/savage \
# ../../data/scratch/tbrucei/TriTrypDB-43_TbruceiTREU927.gff \
# ../../data/intermediate/tbrucei_read_counts/savage \
# ../../data/scratch/sam-to-bam-output/telleria/SRR965341.sorted.bam \
# ../../data/intermediate/tbrucei_read_counts/telleria

# make directory for reads count output from htseq
mkdir -p ../../data/intermediate/tbrucei_read_counts/savage
mkdir -p ../../data/intermediate/tbrucei_read_counts/telleria

# path to bam files
BAM_DIR=$1

# T. brucei genome annotation file.
GFF_FILE=$2

# read counts path
READ_COUNTS_DIR=$3

# telleria data and output dir
TELLERIA_BAM=$4
TELLERIA_OUT=$5

# reads counting (Savage single-end reads)
```

```
for bam_file in ${BAM_DIR}/*.sorted.bam; do
    bam_file_name=$(basename "$bam_file" .sorted.bam)

        htseq-count \
            -f bam \
            -s no \
            -t exon \
            -i Parent \
            $bam_file \
            $GFF_FILE \
            > ${READ_COUNTS_DIR}/${bam_file_name}.counts.txt
done


# process Telleria's paired-end data
telleria_bam_file_name=$(basename "$TELLERIA_BAM" .sorted.bam)

        htseq-count \
            -f bam \
            -r pos \
            -s no \
            -t exon \
            -i Parent \
            $TELLERIA_BAM \
            $GFF_FILE \
            > ${TELLERIA_OUT}/${telleria_bam_file_name}.counts.txt
```

Generating MultiQC report

```
# #!/bin/bash
#
#Script to generate MultiQC reports using the MultiQC tool
#
# USAGE:
# bash generate-multiqc-reports.sh \
# ../../results/figures/fastqc_reports/savage \
# ../../data/scratch/reads-alignment-output/savage \
# ../../data/intermediate/tbrucei_read_counts/savage \
# ../../results/figures/multiqc_reports/savage

# bash generate-multiqc-reports.sh \
# ../../results/figures/fastqc_reports/telleria \
# ../../data/scratch/reads-alignment-output/telleria \
# ../../data/intermediate/tbrucei_read_counts/telleria \
# ../../results/figures/multiqc_reports/telleria

# make directory to store the results
mkdir -p ../../results/figures/multiqc_reports/savage/
mkdir -p ../../results/figures/multiqc_reports/telleria/
```

```
# fastqc directory
FASTQC_DIR=$1

# Hisat2 directory
HISAT2_DIR=$2

# Htseq directory
HTSEQ_DIR=$3

# Multiqc output directory
OUT_DIR=$4

# run mutiqc
multiqc ${FASTQC_DIR}/*_fastqc.zip ${HISAT2_DIR}/*.txt ${HTSEQ_DIR}/*.counts.txt --outdir ${OUT_DIR}
```

Filtering out non-protein coding genes

Before loading the data into R, filter out the non-protein coding genes which include ncRNA, snRNA, snoRNA, pseudogenic transcripts, rRNA and tRNA.

```
# #!/bin/bash
# script to exclude gene features from reads counts

# USAGE:
# ./exclude-features.sh \
# ../../data/intermediate/tbrucei_read_counts/savage \
# ../../data/intermediate/excluded_features.txt \
# ../../data/intermediate/tbrucei_read_counts_mRNA-only/savage \
# ../../data/intermediate/tbrucei_read_counts_mRNA-only/savage-telleria_tbrucei_read_counts_mRNA-only

# ./exclude-features.sh \
# ../../data/intermediate/tbrucei_read_counts/telleria \
# ../../data/intermediate/excluded_features.txt \
# ../../data/intermediate/tbrucei_read_counts_mRNA-only/telleria \
# ../../data/intermediate/tbrucei_read_counts_mRNA-only/savage-telleria_tbrucei_read_counts_mRNA-only

# create directory for the filtered counts
mkdir -p ../../data/intermediate/tbrucei_read_counts_mRNA-only/savage
mkdir -p ../../data/intermediate/tbrucei_read_counts_mRNA-only/telleria

# reads counts directory
READ_COUNTS_DIR=$1

# excludes features file
EXCLUDED_FEAT=$2

# mRNA only output directory
COUNTS_OUT=$3

#combined directory
COMBINED_DIR=$4
```

```
for file in ${READ_COUNTS_DIR}/*.counts.txt; do
  counts_file=$(basename "$file" .counts.txt)
        grep -v -f ${EXCLUDED_FEAT} ${file} > \
            ${COUNTS_OUT}/${counts_file}.counts.txt
done


# copy savage and telleria read counts in a combined directory
# exclude samples which failed quality control (SRR039951, SRR039937, SRR039938)
mkdir -p $COMBINED_DIR

cp $COUNTS_OUT/*.counts.txt $COMBINED_DIR/
rm $COMBINED_DIR/SRR039951.counts.txt $COMBINED_DIR/SRR039937.counts.txt $COMBINED_DIR/SRR039938.counts
```

Analysis in R

Importing samples count data into R

For further analysis, samples read counts are read into R. To read the sample counts data into R using the script below, simply type `source(here::here("scripts","analysis","import-read-counts-into-r.R")` on the R console and hit enter.

```
#!/usr/bin/Rscript

# Take 'all' htseq-count results and melt them in to one big dataframe

#Adapted from: https://wiki.bits.vib.be/index.php/NGS_RNASeq_DE_Exercise.4

# required packages
library(tibble)

# # where are we?
cntdir <- here::here("data", "intermediate", "tbrucei_read_counts_mRNA-only",
                    "savage-telleria_tbrucei_read_counts_mRNA-only")
pat <- ".counts.txt"
hisat2.all <- list.files(path = cntdir,
                         pattern = pat,
                         all.files = TRUE,
                         recursive = FALSE,
                         ignore.case = FALSE,
                         include.dirs = FALSE)

# we choose the 'all' series
myfiles <- hisat2.all
DT <- list()

# read each file as array element of DT and rename the last 2 cols
# we created a list of single sample tables
for (i in 1:length(myfiles) ) {
  infile = paste(cntdir, myfiles[i], sep = "/")
  DT[[myfiles[i]]] <- read.table(infile, header = F, stringsAsFactors = FALSE)
  cnts <- gsub("(.*).counts.txt", "\\1", myfiles[i])
  colnames(DT[[myfiles[i]]]) <- c("ID", cnts)
```

```
}

# merge all elements based on first ID columns
tbrucei_reads_count_raw <- DT[[myfiles[1]]]

# inspect
#head(tbrucei_reads_count_raw)

# we now add each other table with the ID column as key
for (i in 2:length(myfiles)) {
  y <- DT[[myfiles[i]]]
  z <- merge(tbrucei_reads_count_raw, y, by = c("ID"))
  tbrucei_reads_count_raw <- z
}

# ID column becomes rownames
rownames(tbrucei_reads_count_raw) <- tbrucei_reads_count_raw$ID
tbrucei_reads_count_raw <- tbrucei_reads_count_raw[,-1]

## add total counts per sample
tbrucei_reads_count_raw <- rbind(tbrucei_reads_count_raw,
                                 tot.counts=colSums(tbrucei_reads_count_raw))

# inspect and look at the top row names!
#head(tbrucei_reads_count_raw)

#tail(tbrucei_reads_count_raw)

####################################
# take summary rows to a new table
# ( not starting with Tb and tmp with invert=TRUE )

# transpose table for readability
reads_count_summary <- tbrucei_reads_count_raw[grep("^Tb|^tmp", rownames(tbrucei_reads_count_raw),
                                                    perl=TRUE, invert=TRUE), ]

# review
#reads_count_summary

# transpose table
t(reads_count_summary)

# write summary to file
write.csv(reads_count_summary,
          file = here::here("data", "intermediate","tbrucei_reads_count_summary.csv"),
          row.names = FALSE)

####################################
# take all data rows to a new table
reads_count <- tbrucei_reads_count_raw[grep("^Tb|^tmp", rownames(tbrucei_reads_count_raw), perl=TRUE, i[illegible]

# inspect final merged table
#head(reads_count, 3)
```

```
# write data to files
saveRDS(reads_count, file = here::here("data", "intermediate", "tbrucei_reads_count.RDS"))

reads_count <- rownames_to_column(reads_count,"transcript_id")
write.csv(reads_count,
          file = here::here("data", "intermediate", "tbrucei_reads_count.csv"),
          row.names = FALSE)

# cleanup intermediate objects
rm(y, z, i, DT, tbrucei_reads_count_raw)
```

Sample quality check

The quality of the samples is checked before further analysis to check for outlier and batch effects.

```
# load required packages and data
source(here::here("scripts","analysis","libraries.R"))
samples.metadata.clean <- readRDS(here::here("data","raw","samples.metadata.clean.RDS"))
reads_count <- readRDS(file = here::here("data", "intermediate", "tbrucei_reads_count.RDS"))

# Create a DGEList object
counts <- DGEList(reads_count, group = samples.metadata.clean$Tissue)

# check the number of genes with no expression in all samples
table(rowSums(counts$counts==0)==15)
#FALSE  TRUE
# 9184   792

# Filtering non-expressed and lowly-expressed genes.
keep.exprs <- filterByExpr(counts, group=samples.metadata.clean$Sample_Name)
filtered.counts <- counts[keep.exprs,, keep.lib.sizes=FALSE]

# # change transcript ids to corresponding gene ids -----------------------------------------
gtf_file <- import(here::here("data","scratch","tbrucei","TriTrypDB-43_TbruceiTREU927.gtf"))
gene_and_transcript_id <- mcols(gtf_file)[,c("gene_id","transcript_id")]
gene_and_transcript_id <- unique(gene_and_transcript_id)


# replace transcript ids with gene ids as rownames
filtered.counts.tmp <- tibble::rownames_to_column(as.data.frame(filtered.counts$counts),
                                                  "transcript_id")

filtered.counts.tmp$gene_id <- gene_and_transcript_id$gene_id[match(filtered.counts.tmp$transcript_id,
                                                                    gene_and_transcript_id$transcript_i[illegible]

filtered.counts.tmp <- as.data.frame(filtered.counts.tmp) %>% remove_rownames %>%
  column_to_rownames(var = "gene_id")

filtered.counts.tmp$transcript_id <- NULL
filtered.counts$counts <- as.matrix(filtered.counts.tmp)

# obtain logCPM unnormalized for plotting purposes ------------------------------------------
# Here, the norm.factors value is 1 for all samples
```

```r
logcpm.unnorm.counts <- cpm(filtered.counts, log = TRUE, prior.count = 2, normalized.lib.sizes = TRUE)

# Normalize for composition bias using TMM
filtered.counts <- calcNormFactors(filtered.counts, method = 'TMM')

# Convert counts per million per gene to log counts per million for further downstream analysis.
logcpm.norm.counts <- cpm(filtered.counts, log = TRUE, prior.count = 2, normalized.lib.sizes = TRUE)

# use ComBat to remove batch effects
modcombat <- model.matrix(~Tissue, data=samples.metadata.clean)
logcpm.norm.counts.combat <- ComBat(dat=logcpm.norm.counts, batch = samples.metadata.clean$Batch,
                                    mod = modcombat)

# save outputs for later analysis
saveRDS(filtered.counts, here::here("data","intermediate","filtered.counts.RDS"))
saveRDS(logcpm.unnorm.counts, here::here("data","intermediate","logcpm.unnorm.counts.RDS"))
saveRDS(logcpm.norm.counts, here::here("data","intermediate","logcpm.norm.counts.RDS"))
saveRDS(logcpm.norm.counts.combat, here::here("data","intermediate","logcpm.norm.counts.combat.RDS"))
saveRDS(gene_and_transcript_id, here::here("data","intermediate","gene_and_transcript_id.RDS"))

# clean up the environment
rm(gtf_file, filtered.counts.tmp)
```

Weighted gene co-expression analysis

We construct the network at this stage. Also, we write out module genes and their module labels which are required as input by FIRE (Finding Informative Regulatory Elements). Motif prediction was performed online at https://tavazoielab.c2b2.columbia.edu/FIRE/

```r
source(here::here("scripts","analysis","libraries.R"))
source(here::here("scripts","analysis","settings.R"))
logcpm.norm.counts.combat <- readRDS(here::here("data","intermediate","logcpm.norm.counts.combat.RDS"))

########################################################################
## Constructing the network
########################################################################

# obtain the required counts data (WGCNA input)
# WGCNA requires genes to be in columns
network.counts <- t(logcpm.norm.counts.combat)

# determine the soft-thresholding power to use
powers <- c(c(1:10), seq(from = 12, to=20, by=2))
sft <- pickSoftThreshold(network.counts, powerVector = powers, verbose = 5)

# Plot to determine the soft thresholding power to use.

# Scale-free topology fit index as a function of the soft-thresholding power
png(filename = here::here("results","figures","soft-thresholding-power.png"), res =1200, type = "cairo"
    width = 4, height = 4, pointsize = 10)
plot(sft$fitIndices[,1], -sign(sft$fitIndices[,3])*sft$fitIndices[,2],
     xlab="Soft Threshold (power)",
     ylab="Scale Free Topology Model Fit,signed R^2",type="n",
```

```
     main = paste("Scale independence"));
text(sft$fitIndices[,1], -sign(sft$fitIndices[,3])*sft$fitIndices[,2],
     labels=powers,cex=0.9,col="red");

# The red line corresponds to using an R^2 cut-off of h
abline(h=0.80,col="red")
dev.off()

# Mean connectivity as a function of the soft-thresholding power
png(filename = here::here("results","figures","mean-connectivity.png"), res =1200, type = "cairo", units
    width = 4, height = 5, pointsize = 10)
plot(sft$fitIndices[,1], sft$fitIndices[,5],
     xlab="Soft Threshold (power)",
     ylab="Mean Connectivity", type="n",
     main = paste("Mean connectivity"))
text(sft$fitIndices[,1], sft$fitIndices[,5], labels=powers, cex=0.9,col="red")
dev.off()

# construct adjacency matrix
softpower <- 14
adjacency.matrix <- adjacency(network.counts, power=softpower,
                              type = "signed", corFnc = "cor")

# Turn the adjacency matrix to topologicaal overlap matrix to minimize
# the effects of noise and spurious associations
TOM <- TOMsimilarity(adjacency.matrix, TOMType = "signed")
dissTOM <- 1 - TOM

#set diagonal to NA to remove uninformative correlations
diag(adjacency.matrix) <- NA


# Adjacency matrix heatmap plot / network heatmap of selected genes
heatmap_indices <- sample(nrow(adjacency.matrix), 500) # sub-sample for visualization purposes

png(filename = here::here("results","figures","adjacency-matrix-heatmap.png"), res =1200, type = "cairo
    width = 5, height = 5, pointsize = 10)
heatmap.2(t(adjacency.matrix[heatmap_indices, heatmap_indices]),
          col=redgreen(75),
          labRow=NA, labCol=NA,
          trace='none', dendrogram='row',
          xlab='Gene', ylab='Gene',
          main='Adjacency matrix',
          density.info='none', revC=TRUE)
dev.off()



# remove adjacency matrix and TOM to free up memory
rm(adjacency.matrix)
gc()
```

```r
#################################################################
## Detecting co-expression modules in R
#################################################################

# view the dendrogram based on hierachical clustering of genes
gene.tree <- flashClust(as.dist(dissTOM), method = "average")

# plot the gene tree
png(filename = here::here("results","figures","gene-tree.png"), res =1200, type = "cairo", units = 'in'
    width = 7, height = 8, pointsize = 10)
#sizeGrWindow(12,9) #open graphical window
plot(gene.tree, xlab="", sub="", main = "Gene clustering based on TOM dissimilarity",
     labels = FALSE, hang = 0.04)
dev.off()

# identify the modules
module.labels <- cutreeDynamicTree(gene.tree, deepSplit = FALSE,
                                   minModuleSize = 30)

#view
table(module.labels)

# convert labels to colours
module.colours <- labels2colors(module.labels)

# view
table(module.colours)

# a list of 28 modules

# black          blue          brown          cyan      darkgreen
# 438           614           547           219           102
# darkgrey    darkorange       darkred darkturquoise         green
# 93            80           106           100           528
# greenyellow          grey         grey60     lightcyan    lightgreen
# 251            59           191           193           164
# lightyellow       magenta  midnightblue        orange          pink
# 129           264           200            86           383
# purple            red     royalblue        salmon           tan
# 251           460           127           230           243
# turquoise         white        yellow
# 732            61           539

# visualize the gene tree and TOM matrix together using TOM plot
# if necessary, raise dissTOM to a power to make moderately strong connection more visible in heatmap
diag(dissTOM) <- NA

png(filename = here::here("results","figures","gene-tree-and-dissTOM.png"), res =1200, type = "cairo", u
    width = 5, height = 6, pointsize = 10)
TOMplot(dissTOM, gene.tree, as.character(module.colours))
dev.off()

# remove matrix to free memory
```

```
rm(dissTOM)
gc()


# plot gene dendrogram
png(filename = here::here("results","figures","gene-tree-and-colours.png"), res =1200, type = "cairo",
    width = 6, height = 6, pointsize = 10)
#sizeGrWindow(8,6) #open graphical window
plotDendroAndColors(gene.tree, module.colours, "Dynamic Tree Cut", dendroLabels = FALSE,
                    hang = 0.03, addGuide = TRUE, guideHang = 0.05,
                    main = "Gene dendrogram and module colours")
dev.off()

# get hub genes
# choose power 4: https://support.bioconductor.org/p/46342/
module.hub.genes <- chooseTopHubInEachModule(network.counts, module.colours,
                                             power = 4,type = "signed")

# # A list of module hub genes
module.hub.genes
# black              blue            brown              cyan
# "Tb927.7.1790"   "Tb927.8.3620"  "Tb927.11.1570"   "Tb927.2.5530"
# darkgreen          darkgrey       darkorange          darkred
# "Tb927.11.14020"   "Tb927.9.9450"    "Tb927.8.710"   "Tb927.2.5270"
# darkturquoise             green      greenyellow           grey60
# "Tb927.8.6650" "Tb927.10.13790"    "Tb927.7.920"  "Tb927.10.3820"
# lightcyan       lightgreen      lightyellow          magenta
# "Tb927.11.6440"   "Tb927.10.720"  "Tb927.10.2560"   "Tb927.9.6290"
# midnightblue           orange              pink           purple
# "Tb927.11.6640"   "Tb927.9.2520"  "Tb927.10.6200"    "Tb927.1.600"
# red        royalblue           salmon                tan
# "Tb927.7.6920" "Tb927.10.15680"  "Tb927.11.1450"   "Tb927.3.2930"
# turquoise             white           yellow
# "Tb927.9.15630"   "Tb927.8.7980"   "Tb927.1.3550"


##############################################################################
## Network export to cytoscape
##############################################################################

# select modules of interest
all.modules <- c('black', 'cyan', 'grey','brown',
                         'midnightblue','blue','darkgreen','tan', 'darkgrey','darkorange',
                         'darkred','darkturquoise','green','greenyellow','grey60','lightcyan',
                         'lightgreen','lightyellow','magenta','orange','pink','purple','red',
                         'royalblue','salmon','turquoise','white','yellow') # all module colours

# enriched modules
enriched.modules <- c("black","tan","brown","blue","turquoise","magenta","darkturquoise",
                      "green","red","pink","salmon","lightyellow","purple","greenyellow")

# obtain gene ids
gene.ids <- rownames(logcpm.norm.counts.combat)
```

```
# select module genes
inModules <- is.finite(match(module.colours, all.modules)) # whole network modules
#inModules <- is.finite(match(module.colours, enriched.modules)) # enriched modules

modGenes <- gene.ids[inModules]

# select the corresponding dissTOM based on module genes
modTOM <- TOM[inModules, inModules]
dimnames(modTOM) <- list(modGenes, modGenes)

# Export the network into edge and node list files Cytoscape can read
exportNetworkToCytoscape(modTOM,
                         edgeFile = here::here("data","intermediate","cytoscape_input_files",
                                               "CytoscapeInput-edges_whole_network_thresh0.3.txt"),
                         nodeFile = here::here("data","intermediate","cytoscape_input_files",
                                               "CytoscapeInput-nodes_whole_network_thresh0.3.txt"),

                         weighted = TRUE,
                         threshold = 0.3,
                         nodeNames = modGenes,
                         nodeAttr = module.colours[inModules]);

# network modules
# create a dataframe with node attributes
enriched.module.colours <- module.colours[inModules] #get enriched module colours from module.colours
node.attributes <- cbind(modGenes, module=module.colours) # get node attr. for whole network
# node.attributes <- cbind(modGenes, module=enriched.module.colours) # node atrr. for enriched modules

node.attributes <- as.data.frame(node.attributes)

# Add RGB versions of colour modules
node.attributes$colourRGB <- col2hex(node.attributes$module)

# write out a node attributes files with hexadecimal colour names for module genes
write.table(node.attributes,
            file = here::here("data","intermediate","cytoscape_input_files",
                              "Cytoscape_node_attributes_whole_network_thresh0.3.txt"),
            row.names = FALSE,
            quote = FALSE, sep = "\t")

########################################
## FIRE Motif Prediction Input
########################################
# write out cluster/module genes and their corresponding module labels for use by
# FIRE (Finding Informative Regulatory Elements)

fire.clusters.colours <- data.frame(modGenes, module.labels[inModules], module.colours[inModules])

# sort by module labels; FIRE input should start from 0 in module labels column.
fire.clusters.colours <- fire.clusters.colours[order(fire.clusters.colours$module.labels.inModules.),
                                               c(1,2,3)]

# rename columns
```

```
colnames(fire.clusters.colours) <- c("gene", "label","colour")

write.table(as.data.frame(fire.clusters.colours), file = here::here("data","intermediate","tbrucei_FIRE_
            quote = FALSE, row.names = FALSE, sep = "\t")

# Also, write out module genes in a text file.
write.table(data.frame(modGenes),
            file = here::here("data","intermediate","tbrucei_module_genes.txt"),
            row.names = FALSE,
            quote = FALSE,
            col.names = FALSE)


saveRDS(gene.tree, file = here::here("data","intermediate","gene.tree.RDS"))
saveRDS(all.modules, file = here::here("data","intermediate","all.modules.RDS"))
saveRDS(module.colours, file = here::here("data","intermediate","module.colours.RDS"))
saveRDS(module.hub.genes, file = here::here("data","intermediate","module.hub.genes.RDS"))
```

Functional Analysis

Code used in functional analysis was adopted from https://github.com/elsayed-lab/manuscript-shared-rnaseq

Loading annotations from the packages

```
source(here::here("scripts","analysis","libraries.R"))
source(here::here("scripts","analysis","settings.R"))
source(here::here("scripts","utils","enrichment_analysis.R"))
source(here::here("scripts","utils","annotations.R"))
source(here::here("scripts","utils","wgcna.R"))
source(here::here("scripts","utils","util.R"))
logcpm.norm.counts.combat <- readRDS(here::here("data","intermediate","logcpm.norm.counts.combat.RDS"))
gene_and_transcript_id <- readRDS(here::here("data","intermediate","gene_and_transcript_id.RDS"))
gene.tree <- readRDS(file = here::here("data","intermediate","gene.tree.RDS"))
all.modules <- readRDS(file = here::here("data","intermediate","all.modules.RDS"))
module.colours <- readRDS(file = here::here("data","intermediate","module.colours.RDS"))
module.hub.genes <- readRDS(here::here("data","intermediate","module.hub.genes.RDS"))

#################################################################
# load gene annotations from packages
#################################################################
orgdb <- get("Trypanosoma.brucei.TREU927")

# Fix AnnotationDbi namespace mess
assign('select', dplyr::select, envir=.GlobalEnv)
assign('get',    base::get, envir=.GlobalEnv)

gene_info <- load_parasite_annotations(orgdb, rownames(logcpm.norm.counts.combat),
                                       keytype="GID")

# add the transcript id column to the gene_info
gene_info$transcript_id <- gene_and_transcript_id$transcript_id[match(gene_info$gene_id,
```

```
                                                                gene_and_transcript_id$gene_id)]

# Get transcript lengths (sum of all exon lengths for each gene)
txdb <- orgdb@txdbSlot
transcript_lengths <- transcriptLengths(txdb)
transcript_lengths <- transcript_lengths[transcript_lengths$tx_name %in%
                                          gene_info$transcript_id,]

# add the transcript lengths to gene_info
gene_info[match(transcript_lengths$tx_name, gene_info$transcript_id),
          'transcript_length'] <- transcript_lengths$tx_len
gene_info$transcript_length <- as.numeric(gene_info$transcript_length)

# A gene was excluded in the annotation database as a result of an orphan transcript.
#Add its placeholder.
## ---not run---
# gene_info <- rbind(gene_info, data.frame(gene_id='Tb927.4.4663',
#                                           chromosome='4',
#                                           description=NA, strand=NA, type=NA,
#                                           transcript_length=NA))


# Keep only the feature information remaining genes
gene_info <- gene_info[gene_info$gene_id %in% rownames(logcpm.norm.counts.combat),] #WGCNA input counts

# For now, just grab the description for the first transcript
#gene_info <- gene_info[!duplicated(gene_info$gene_id),]

# Gene IDs
gene_ids <- rownames(logcpm.norm.counts.combat)

# gene annotations preview
kable(head(gene_info), caption='Preview of gene annotations.')

#########################################################################################
# load GO terms associated with each parasite gene
#########################################################################################

# # load go terms from annotation package
# go_terms <- load_go_terms(orgdb, rownames(logcpm.norm.counts.combat),
#                            keytype='GID')
#
# # this take time to run, so save it to avoid re-running.
# saveRDS(go_terms, file = here::here("data","intermediate","go_terms.RDS"))
go_terms <- readRDS(file = here::here("data","intermediate","go_terms.RDS"))

# Exclude genes not found in count table --not run--
#go_terms <- go_terms[go_terms$GID %in% rownames(logcpm.norm.counts.combat),]

# gene / go term mapping
gene_go_mapping <- as.data.frame(unique(go_terms %>% select(GID, GO, ONTOLOGY)))
colnames(gene_go_mapping) <- c('gene', 'category', 'ontology')
```

```r
# go id / term mapping
go_term_id_mapping <- as.data.frame(unique(go_terms[c('GO', 'TERM', 'ONTOLOGY')]))
colnames(go_term_id_mapping) <- c("category", "term", "ontology")

##########################################################################################
# Load KEGG annotations
##########################################################################################

gene_kegg_mapping <- load_kegg_mapping(orgdb, rownames(logcpm.norm.counts.combat),
                                       keytype="GID")

kegg_pathways <- load_kegg_pathways(orgdb, rownames(logcpm.norm.counts.combat),
                                    keytype="GID")


# Rename gene/KEGG mapping columns to be consistent with GO mapping
colnames(gene_kegg_mapping) <- c('gene', 'category')
colnames(kegg_pathways)     <- c('category', 'name', 'class', 'description')

kegg_pathways <- unique(kegg_pathways)


################################################
# GO Enrichment
################################################

# get the number of modules
num_modules <- length(unique(module.colours))

# Create gene lengths vector
gene_lengths <- gene_info$transcript_length
names(gene_lengths) <- gene_info$gene_id

# save the module sizes
# Data frame of module sizes
module_counts <- c()
for (color in unique(module.colours)) {
  module_counts <- append(module_counts, sum(module.colours == color))
}

# create a mapping from module id to number of genes for later use
module_sizes <- data.frame(module_id=unique(module.colours),
                           num_genes=module_counts)

# Initialize parallelization
cl <- makeCluster(max(1, min(10, detectCores() - 2, na.rm = TRUE)))
registerDoParallel(cl)
message("Performing GO enrichment")

# Check each module for enrichment in GO terms and save result in a list
module_go_enrichment <- foreach(color=unique(module.colours), .packages=c('goseq')) %dopar% {
  set.seed(1)
  # Measure GO enrichment for module
```

```r
  enriched <- tryCatch({
    # module gene ids
    in_module_geneids <- gene_ids[module.colours == color]
    message(sprintf("[GO enrichment] %s", color))

    # T. brucei GO enrichment
    enriched <- test_gene_enrichment(in_module_geneids, gene_ids,
                                     gene_go_mapping, gene_lengths)

    # Add descriptions
    enriched <- merge(enriched, go_term_id_mapping, by='category')
  }, error=function(e) {
    # goseq fails in some cases; have not been able to track down cause yet
    # to avoid errors we will just return an empty result set
    warning(sprintf("GO enrichment failed for module %s", color))
    cbind(
      get_enrichment_placeholder(),
      term=numeric(0),
      ontology=numeric(0)
    )
  })
  enriched
}
names(module_go_enrichment) <- unique(module.colours)

# remove any null/empty entries from the results
module_go_enrichment <- module_go_enrichment[!sapply(module_go_enrichment, is.null)]

# unregister cpus
stopCluster(cl)

# save the GO enrichment results
saveRDS(module_go_enrichment, file = here::here("data","intermediate","module_go_enrichment.RDS"))


#--------------------------------------
# Print GO enrichment results
#--------------------------------------
# temporarily repeat the gene / go term mapping to add 'term' column
gene_go_mapping_tmp <- as.data.frame(unique(go_terms %>% select(GID, GO, TERM, ONTOLOGY)))
colnames(gene_go_mapping_tmp) <- c('gene', 'category', 'term', 'ontology')

gene_info_tmp <- gene_info %>% select(-chromosome, -strand,
                                       - type, -transcript_length)
colnames(gene_info_tmp) <- c("gene","description")

tmp <- cbind(gene_info_tmp, color=module.colours)

#tmp <- cbind(gene=gene_ids, color=module.colours)
gene_mapping <- merge(gene_go_mapping_tmp, tmp, by='gene')
cat(sprintf('- Total enriched modules: %d\n',
            sum(sapply(module_go_enrichment, nrow) > 0)))
```

```r
# create tables of the results in this document
print_enrichment_results(module_go_enrichment, module_sizes, 'GO terms',
                         NULL, gene_mapping,
                         output_dir=here::here("results","tables"),
                         enrichment_type='go',
                         include_gene_lists=FALSE)

enriched_colors_go <- get_enriched_modules(module_go_enrichment)

# Module enrichment status (used in dendrogram plots)
go_enrichment_status   <- as.numeric(module.colours %in% enriched_colors_go)


saveRDS(gene_mapping, file = here::here("data","intermediate","gene_mapping.RDS"))

################################################
# KEGG Enrichment
################################################

# Check each module for enrichment in KEGG terms and save result in a list
cl <- makeCluster(max(1, min(10, detectCores() - 2, na.rm = TRUE)))
registerDoParallel(cl)

message("Performing KEGG enrichment")

# Check each module for enrichment in GO terms and save result in a list
module_kegg_enrichment <- foreach(color=unique(module.colours), .packages=c('goseq')) %dopar% {
  set.seed(1)

  # Measure KEGG enrichment for module
  enriched <- tryCatch({
    in_module_geneids <- gene_ids[module.colours == color]
    enriched <- test_gene_enrichment(in_module_geneids, gene_ids,
                                     gene_kegg_mapping, gene_lengths)

    enriched <- unique(merge(enriched, kegg_pathways[,c('category','name')],
                             by='category'))
  }, error=function(e) {
    # goseq fails in some cases; have not been able to track down cause yet
    warning(sprintf("KEGG enrichment failed for module %s", color))
    return(get_enrichment_placeholder())
  })
  enriched
}

names(module_kegg_enrichment) <- unique(module.colours)

# remove any null/empty entries from the results
module_kegg_enrichment <- module_kegg_enrichment[!sapply(module_kegg_enrichment, is.null)]

# unregister cpus
stopCluster(cl)
```

```r
# save the KEGG enrichment results
saveRDS(module_kegg_enrichment, file = here::here("data","intermediate","module_kegg_enrichment.RDS"))


#------------------------------------
# Print KEGG enrichment results
#------------------------------------

cat(sprintf('- Total enriched modules: %d\n',
            sum(sapply(module_kegg_enrichment, nrow) > 0)))

# create tables of the results in this document --function has bugs for kegg enrichment type--
# print_enrichment_results(module_kegg_enrichment, module_sizes,
#                          'KEGG pathway',
#                          output_dir= here::here("results","tables"),
#                          enrichment_type='kegg')

enriched_colors_kegg <- get_enriched_modules(module_kegg_enrichment)


# Module enrichment status (used in dendrogram plots)
kegg_enrichment_status <- as.numeric(module.colours %in% enriched_colors_kegg)


# add enrichment information to the Dendrogram
unassigned_modules <- as.numeric(module.colours == 'grey')

png(filename = here::here("results","figures","gene-tree-and-module-enrichment-status.png"), res =1200,
    type = "cairo", units = 'in', width = 6, height = 6, pointsize = 10)
WGCNA::plotDendroAndColors(gene.tree,
                           cbind(module.colours, go_enrichment_status,
                                 kegg_enrichment_status, unassigned_modules),
                           groupLabels=c(sprintf("Modules\n(n=%s)", num_modules),
                                         #sprintf("Red = upregulated at %s", CONFIG$de_cond2),
                                         "GO enrichment", "KEGG enrichment", "Unassigned"),
                           #cex.colorLabels=cex_color_labels, cex.main=cex_main,
                           # cex.axis=cex_axis, cex.lab=cex_lab,
                           dendroLabels=FALSE,
                           #marAll=c(4,8,6,4),
                           guideHang=0.05)
dev.off()


################################################################
### Save results
################################################################

# Save each module's enrichment results as csv files
module_output_dir <- here::here("results","tables","enrichment_results")
if (!dir.exists(module_output_dir)) {
  dir.create(module_output_dir, recursive=TRUE)
}
```

```r
# GO enrichment
output_module_enrichment_results(module_go_enrichment, module_output_dir,
                                 'go', go_term_id_mapping)

# KEGG enrichment
output_module_enrichment_results(module_kegg_enrichment, module_output_dir,
                                 'kegg', kegg_pathways %>% select(-description))

################################################################################
# Save GO enrichment results

# get only the enriched go modules
enriched_module_go_enrichment <- module_go_enrichment[names(module_go_enrichment) %in% enriched_colors_g

write.xlsx(enriched_module_go_enrichment,
           file = here::here("results","tables","modules_go_enrichment_results.xlsx"))

###################################################################################
#save KEGG enrichment results

# get only the enriched kegg modules
enriched_module_kegg_enrichment <- module_kegg_enrichment[names(module_kegg_enrichment) %in% enriched_co

write.xlsx(enriched_module_kegg_enrichment,
           file = here::here("results","tables","modules_kegg_enrichment_results.xlsx"))


################################################################################
# Create co-expression results data frame
result <- cbind(gene_info, color=module.colours)

# drop unneeded columns
keep_cols <- intersect(c('gene_id', 'color', 'description', 'chromosome',
                         'strand', 'transcript_length'), colnames(result))
result <- tbl_df(result[,keep_cols])

# add expression-related fields
result$expr_variance <- apply(logcpm.norm.counts.combat, 1, var)
result$expr_mean <- apply(logcpm.norm.counts.combat, 1, mean)

# replace colors with numbers
#module.colours <- module_label_mapping$number[match(module.colours, module_label_mapping$color)]

#result$color <- module_label_mapping$number[match(result$color, module_label_mapping$color)]

# Write out an excel/csv file for the result dataframe
openxlsx::write.xlsx(result, file=here::here("results","tables","coexpression_network_genes.xlsx"))


####################################################################################
# save module hub genes domain description
module_hub_genes_description <- gene_info %>%
                filter(gene_id %in% as.data.frame(module.hub.genes)$module.hub.genes) %>%
```

```
                 select(gene_id, description)

module_hub_genes_df <- as.data.frame(module.hub.genes)

module_hub_genes_df <- tibble::rownames_to_column(module_hub_genes_df, var="module")

module_hub_genes_domain_description <- merge(module_hub_genes_df, module_hub_genes_description,
                                             by.x="module.hub.genes", by.y="gene_id")

# reorder and rename columns
module_hub_genes_domain_description <- module_hub_genes_domain_description[,c(2,1,3)]

module_hub_genes_domain_description <- module_hub_genes_domain_description %>%
  dplyr::rename(hub_gene=module.hub.genes)

openxlsx::write.xlsx(module_hub_genes_domain_description,
           file = here::here("results","tables","hub_genes.xlsx"))

#########################################################################################
# Add hub genes and description to top GO terms in each module

modules_top_over_represented_go_terms <-readxl::read_excel(here::here("results",
                                                                       "tables",
                                                                       "modules_top_over_represented_go_

enriched_modules_hub_genes <- module_hub_genes_domain_description %>%
              filter(module %in% modules_top_over_represented_go_terms$module)

modules_top_over_represented_go_terms_and_hub_genes <- merge(modules_top_over_represented_go_terms,
                                                              enriched_modules_hub_genes, by="module")

openxlsx::write.xlsx(modules_top_over_represented_go_terms_and_hub_genes,
           file = here::here("results",
                             "tables",
                             "modules_top_over_represented_go_terms_and_hub_genes.xlsx"))
```
