# Supplementary material for: Gene co-expression network analysis of Trypanosoma brucei in tsetse fly vector
Source: Parasit Vectors. 2021 Jan 22;14:74. doi: 10.1186/s13071-021-04597-6 (PMC7821691; doi:10.1186/s13071-021-04597-6)
Supplement: Supplementary file 2 — Additional file 2: Table S1. Sample metadata for samples used in this study. Samples highlighted in red were excluded from analysis because of failing quality assessment [file 13071_2021_4597_MOESM2_ESM.docx]

|  | Library Name | Run | Sample Name | Tissue | Source Study |
| --- | --- | --- | --- | --- | --- |
| 1 | mg1 | SRR039378 | MG1 | MG | Savage et al. (2016) |
| 2 | mg1 | SRR039381 | MG1 | MG | Savage et al. (2016) |
| 3 | mg1 | SRR039453 | MG1 | MG | Savage et al. (2016) |
| 4 | MG2_SL | SRR039454 | MG2 | MG | Savage et al. (2016) |
| 5 | MG2_SL | SRR039455 | MG2 | MG | Savage et al. (2016) |
| 6 | PV2_SL | SRR039456 | PV2 | PV | Savage et al. (2016) |
| 7 | PV2_SL | SRR039457 | PV2 | PV | Savage et al. (2016) |
| 8 | SA2_SL | SRR039939 | SA2 | SG | Savage et al. (2016) |
| 9 | SA2_SL | SRR039940 | SA2 | SG | Savage et al. (2016) |
| 10 | mg1 | SRR039948 | MG1 | MG | Savage et al. (2016) |
| 11 | MG2_SL | SRR039949 | MG2 | MG | Savage et al. (2016) |
| 12 | PV2_SL | SRR039950 | PV2 | PV | Savage et al. (2016) |
| 13 | SA2_SL | SRR039952 | SA2 | SG | Savage et al. (2016) |
| 14 | PV2_SL | SRR042429 | PV2 | PV | Savage et al. (2016) |
| 15 | SA1 | SRR965341 | SA1 | SG | Telleria et al. (2014) |
| 16 | SA1 | SRR039950 | SA1 | SG | Savage et al. (2016) |
| 17 | SA1 | SRR039937 | SA1 | SG | Savage et al. (2016) |
| 18 | SA1 | SRR039938 | SA1 | SG | Savage et al. (2016) |

**Table S1** Samples metadata for samples used in this study. Samples highlighted in red were excluded from analysis because of failing quality assessment.
