# Supplementary figures and images for: Gene co-expression network analysis of Trypanosoma brucei in tsetse fly vector
Source: Parasit Vectors. 2021 Jan 22;14:74. doi: 10.1186/s13071-021-04597-6 (PMC7821691; doi:10.1186/s13071-021-04597-6)

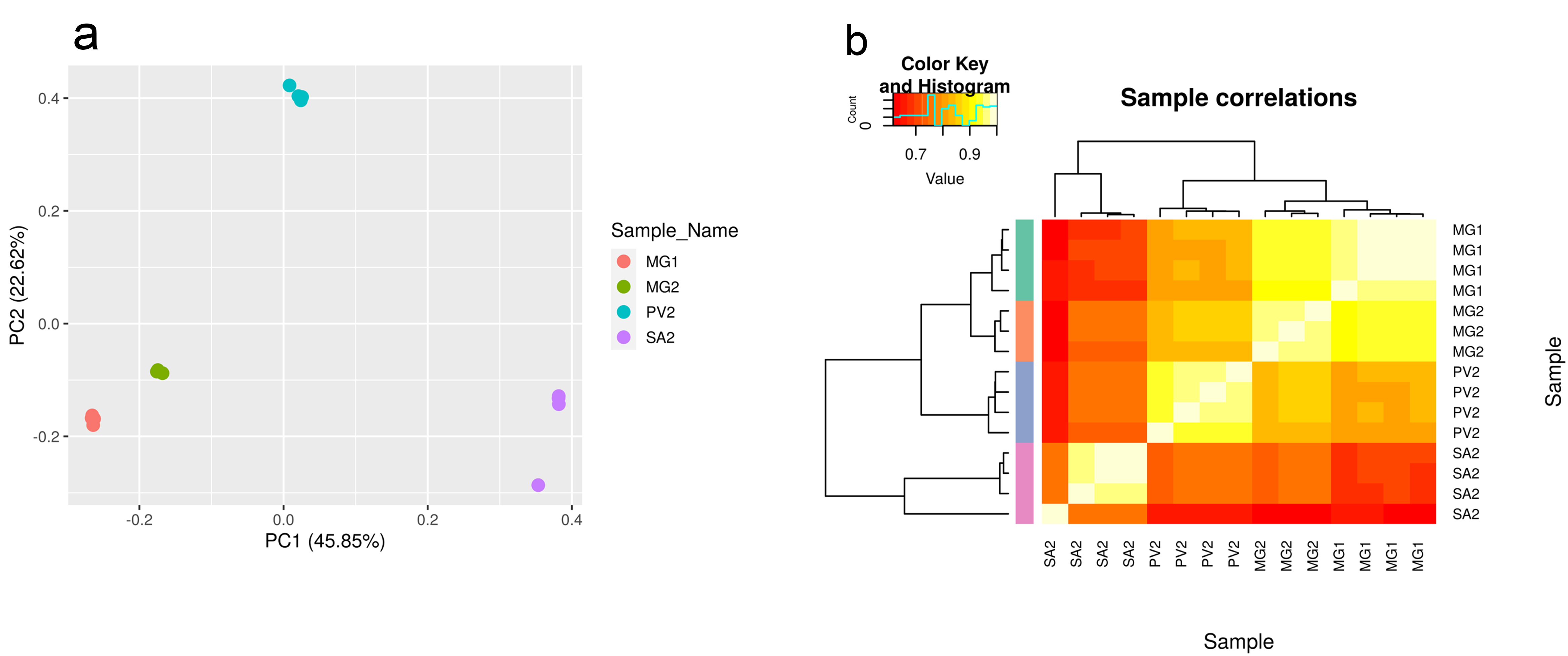

Supplement: Supplementary file 3 — Additional file 3: Figure S1. Principal component analysis (PCA) and Pearson correlation heatmap prior to accounting for batch effects. (a) Each point in the PCA plot represents an experimental sample, and point color indicates a batch that consists of the biological replicates. (b) Color codes along the left side of the sample correlation heatmap indicate samples based on the batch they belong to. MG1 and MG2 are midgut samples, PV2 are proventriculus samples, and SA2 are salivary gland samples [file 13071_2021_4597_MOESM3_ESM.tif]

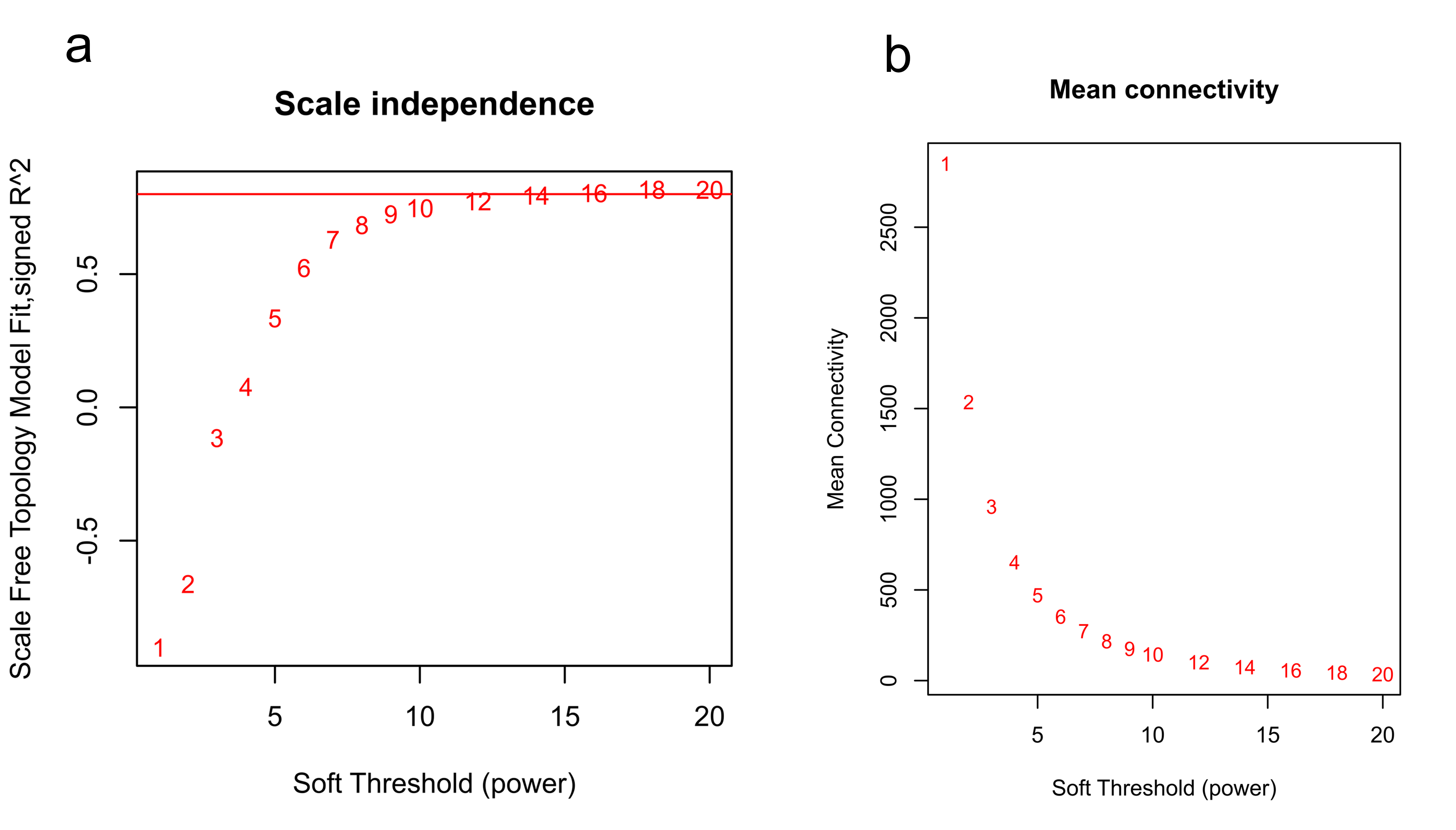

Supplement: Supplementary file 5 — Additional file 5: Figure S3. Scale-free topology plot for selecting the power β for the signed correlation network. (a) Scale free topology index (y axis) as a function of powers, β, 1 to 20 (x axis). (b) Mean connectivity (y axis) as a decreasing function of powers β (x axis) [file 13071_2021_4597_MOESM5_ESM.tif]

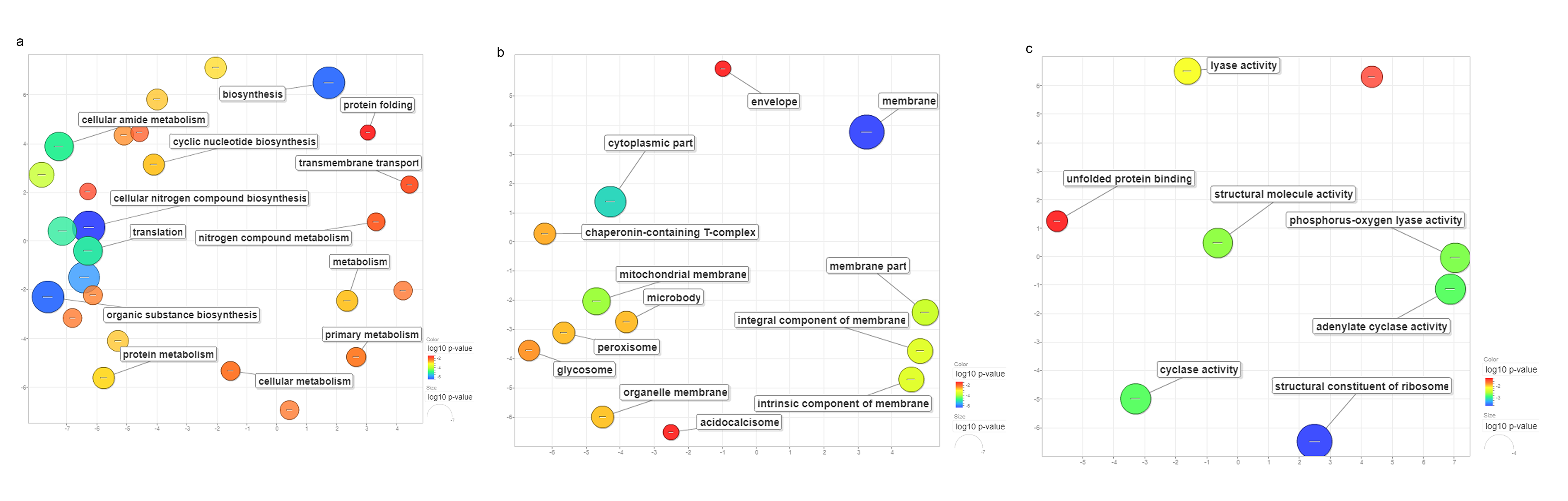

Supplement: Supplementary file 10 — Additional file 10: Figure S4. Black module over-represented GO terms. (a) Biological process GO terms; (b) cellular component GO terms; (c) molecular function GO terms [file 13071_2021_4597_MOESM10_ESM.tif]

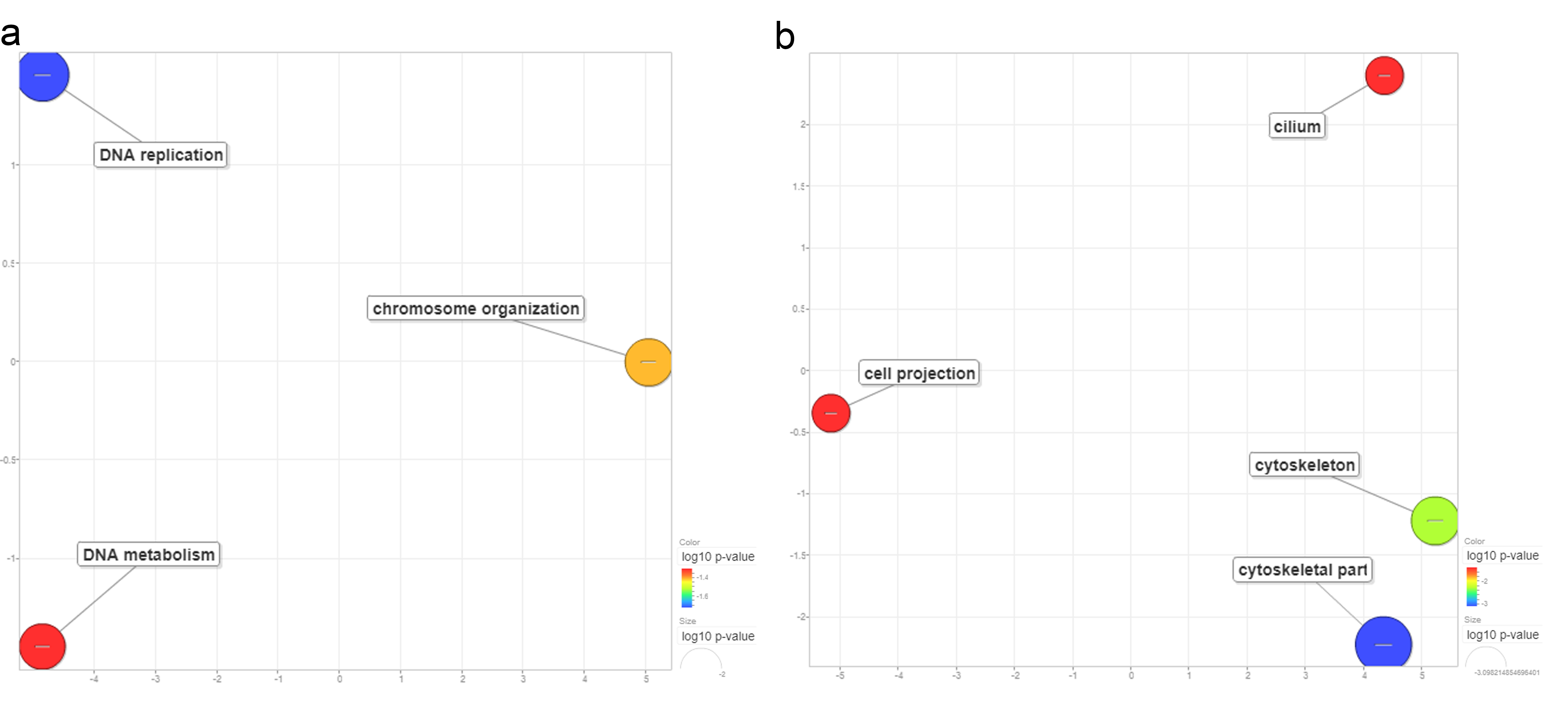

Supplement: Supplementary file 11 — Additional file 11: Figure S5. Red module over-represented GO terms. (a) Biological process GO terms; (b) cellular component GO terms [file 13071_2021_4597_MOESM11_ESM.tif]

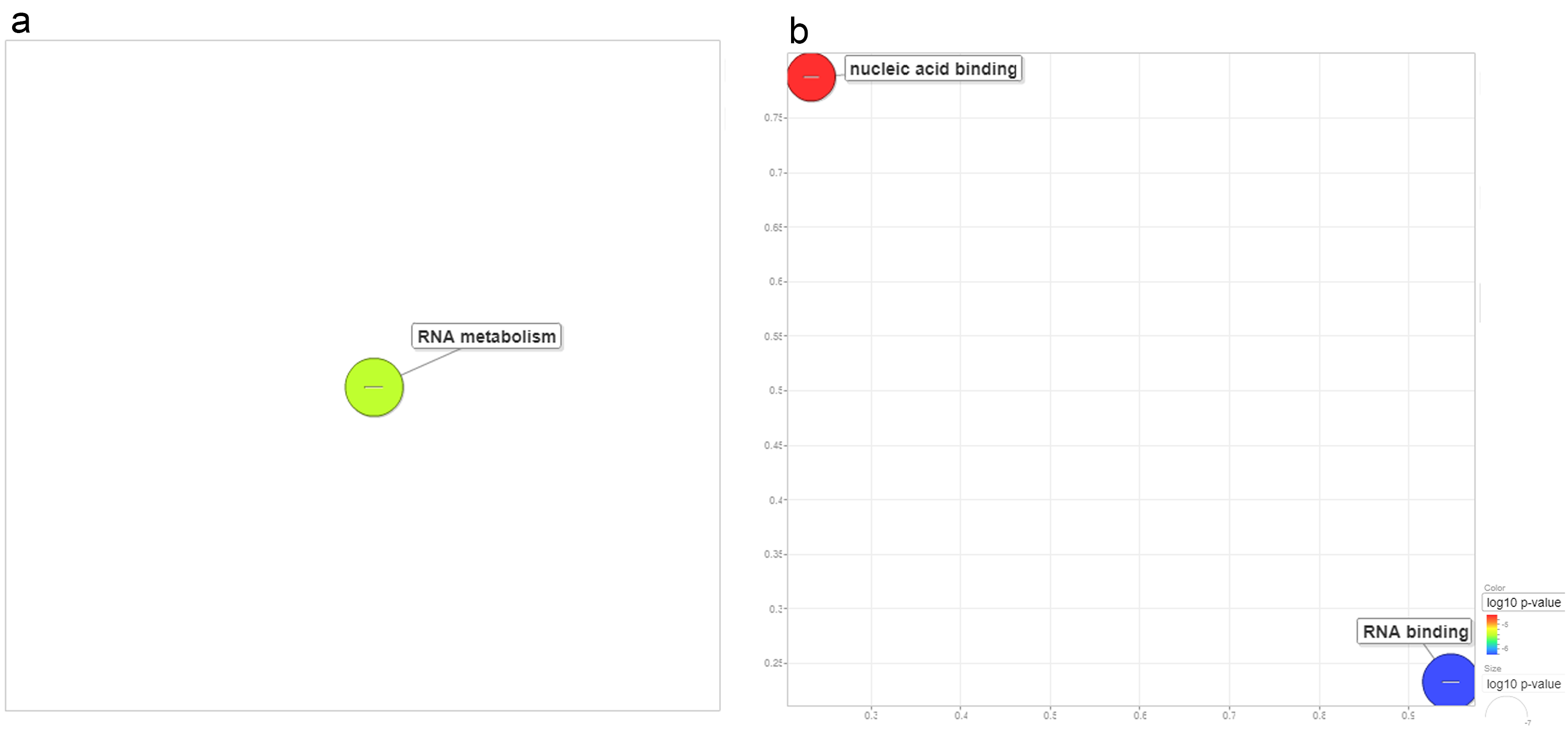

Supplement: Supplementary file 12 — Additional file 12: Figure S6. Salmon module over-represented GO terms. (a) Biological process GO terms; (b) molecular function GO terms [file 13071_2021_4597_MOESM12_ESM.tif]

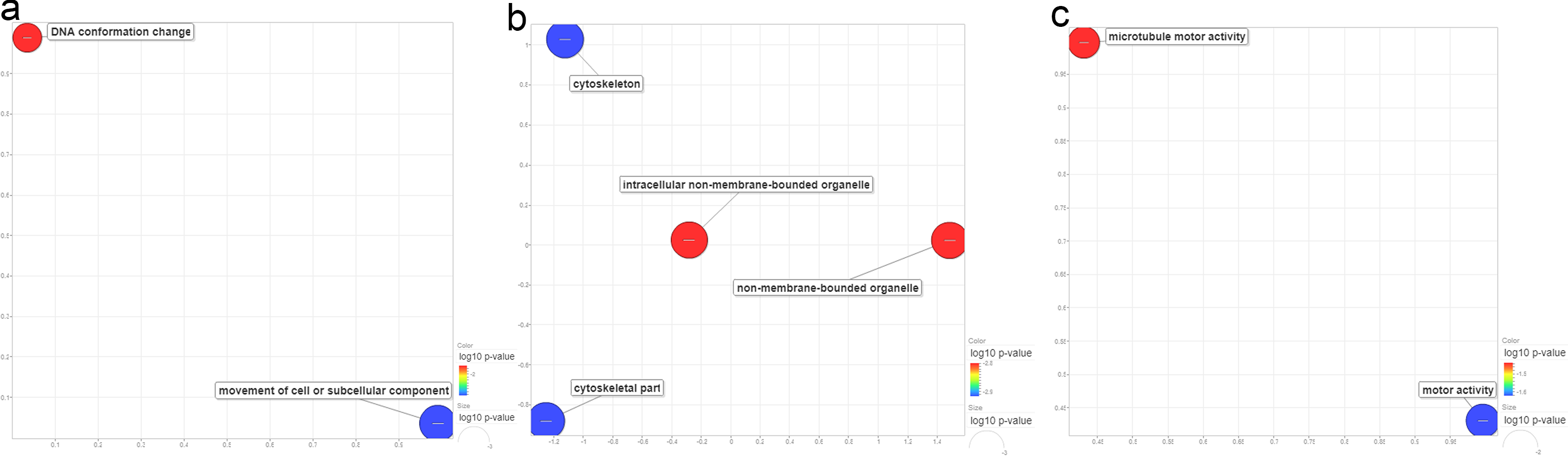

Supplement: Supplementary file 13 — Additional file 13: Figure S7. Greenyellow module over-represented GO terms. (a) Biological process GO terms; (b) cellular component GO terms; (c) molecular function GO terms [file 13071_2021_4597_MOESM13_ESM.tif]
