# Supplementary material for: Gene co-expression network analysis of Trypanosoma brucei in tsetse fly vector
Source: Parasit Vectors. 2021 Jan 22;14:74. doi: 10.1186/s13071-021-04597-6 (PMC7821691; doi:10.1186/s13071-021-04597-6)
Supplement: Supplementary file 7 — Additional file 7: Table S3. Module GO enrichment results; 14 modules with their significantly over- and under-represented GO terms are tabulated [file 13071_2021_4597_MOESM7_ESM.pdf]

Table S3: Module GO Enrichment

a) Lightyellow enrichment (129 genes)

Over-represented GO terms:

category	num_in_subset	num_total	adj_pval	term	ontology
GO:0003735	27	161	0.0000000	structural constituent of ribosome	MF
GO:0005198	27	190	0.0000000	structural molecule activity	MF
GO:0005840	30	260	0.0000000	ribosome	CC
GO:0006412	28	243	0.0000000	translation	BP
GO:0043043	28	245	0.0000000	peptide biosynthetic process	BP
GO:0006518	28	252	0.0000000	peptide metabolic process	BP
GO:0043604	28	259	0.0000000	amide biosynthetic process	BP
GO:0043603	28	268	0.0000000	cellular amide metabolic process	BP
GO:0009058	40	673	0.0000001	biosynthetic process	BP
GO:1901566	31	381	0.0000001	organonitrogen compound biosynthetic process	BP
GO:0044249	38	648	0.0000005	cellular biosynthetic process	BP
GO:0044271	32	476	0.0000010	cellular nitrogen compound biosynthetic process	BP
GO:1901576	37	651	0.0000015	organic substance biosynthetic process	BP
GO:0034645	30	433	0.0000034	cellular macromolecule biosynthetic process	BP
GO:0009059	30	438	0.0000038	macromolecule biosynthetic process	BP
GO:0044391	15	142	0.0002226	ribosomal subunit	CC
GO:1990904	16	192	0.0016499	ribonucleoprotein complex	CC
GO:0034641	39	963	0.0018397	cellular nitrogen compound metabolic process	BP
GO:1901564	37	903	0.0018414	organonitrogen compound metabolic process	BP

GO:0015934	10	82	0.0041045	large ribosomal subunit	CC
GO:0044237	52	1593	0.0045776	cellular metabolic process	BP
GO:0019538	31	763	0.0119541	protein metabolic process	BP
GO:0044267	29	673	0.0122762	cellular protein metabolic process	BP
GO:0006091	7	60	0.0207513	generation of precursor metabolites and energy	BP
GO:0010467	35	891	0.0207513	gene expression	BP
GO:0019843	4	11	0.0207513	rRNA binding	MF
GO:0008152	61	2137	0.0226994	metabolic process	BP
GO:0044238	48	1594	0.0462493	primary metabolic process	BP

b) Magenta enrichment (264 genes)

Over-represented GO terms:

category	num_in_subset	num_total	adj_pval	term	ontology
GO:0005856	54	693	0.0004069	cytoskeleton	CC
GO:0005930	31	293	0.0004069	axoneme	CC
GO:0005929	89	1558	0.0023548	cilium	CC
GO:0042995	89	1558	0.0023548	cell projection	CC
GO:0120025	89	1558	0.0023548	plasma membrane bounded cell projection	CC
GO:0004054	3	3	0.0265477	arginine kinase activity	MF
GO:0016775	3	3	0.0265477	phosphotransferase activity, nitrogenous group as acceptor	MF
GO:0043228	72	1305	0.0265477	non-membrane-bounded organelle	CC
GO:0043232	72	1305	0.0265477	intracellular non-membrane-bounded organelle	CC

c) Greenyellow enrichment (251 genes)

Over-represented GO terms:

category	num_in_subset	num_total	adj_pval	term	ontology
GO:0005856	57	693	0.0012162	cytoskeleton	CC
GO:0006928	21	125	0.0025244	movement of cell or subcellular component	BP
GO:0015630	35	344	0.0025244	microtubule cytoskeleton	CC
GO:0043228	75	1305	0.0025244	non-membrane-bounded organelle	CC
GO:0043232	75	1305	0.0025244	intracellular non-membrane-bounded organelle	CC
GO:0071103	7	22	0.0224329	DNA conformation change	BP
GO:0003774	15	77	0.0253184	motor activity	MF
GO:0003777	14	72	0.0378001	microtubule motor activity	MF
GO:0005875	14	82	0.0378001	microtubule associated complex	CC

d) Black enrichment (438 genes)

Over-represented GO terms:

category	num_in_subset	num_total	adj_pval	term	ontology
GO:0044271	68	476	0.0000002	cellular nitrogen compound biosynthetic process	BP
GO:0016020	92	812	0.0000004	membrane	CC
GO:1901576	80	651	0.0000007	organic substance biosynthetic process	BP
GO:0009058	81	673	0.0000011	biosynthetic process	BP
GO:0044249	78	648	0.0000020	cellular biosynthetic process	BP
GO:0043043	42	245	0.0000059	peptide biosynthetic process	BP
GO:0043604	43	259	0.0000073	amide biosynthetic process	BP

GO:1901566	54	381	0.0000084	organonitrogen compound biosynthetic process	BP
GO:0006412	41	243	0.0000088	translation	BP
GO:0006518	42	252	0.0000088	peptide metabolic process	BP
GO:0043603	43	268	0.0000124	cellular amide metabolic process	BP
GO:0031966	34	212	0.0001033	mitochondrial membrane	CC
GO:0005740	34	216	0.0001587	mitochondrial envelope	CC
GO:1901564	89	903	0.0002295	organonitrogen compound metabolic process	BP
GO:0003735	30	161	0.0002896	structural constituent of ribosome	MF
GO:0016021	47	394	0.0004946	integral component of membrane	CC
GO:0031224	47	394	0.0004946	intrinsic component of membrane	CC
GO:0101031	6	9	0.0005169	chaperone complex	CC
GO:0019538	76	763	0.0016904	protein metabolic process	BP
GO:0004016	11	43	0.0021100	adenylate cyclase activity	MF
GO:0009975	11	43	0.0021100	cyclase activity	MF
GO:0009187	12	52	0.0022011	cyclic nucleotide metabolic process	BP
GO:0009190	12	52	0.0022011	cyclic nucleotide biosynthetic process	BP
GO:0044267	69	673	0.0022011	cellular protein metabolic process	BP
GO:0044281	35	280	0.0022011	small molecule metabolic process	BP
GO:0008152	168	2137	0.0022018	metabolic process	BP
GO:0016849	12	53	0.0025243	phosphorus-oxygen lyase activity	MF
GO:0005198	30	190	0.0029241	structural molecule activity	MF
GO:0005777	24	158	0.0029241	peroxisome	CC
GO:0042579	24	158	0.0029241	microbody	CC
GO:0005832	5	8	0.0035998	chaperonin-containing T-complex	CC
GO:0031090	38	309	0.0044287	organelle membrane	CC
GO:0020015	23	154	0.0051638	glycosome	CC

GO:0044238	130	1594	0.0055108	primary metabolic process	BP
GO:0008150	216	2959	0.0057668	biological_process	BP
GO:0055085	19	118	0.0057668	transmembrane transport	BP
GO:0034645	49	433	0.0059436	cellular macromolecule biosynthetic process	BP
GO:0005743	20	120	0.0062621	mitochondrial inner membrane	CC
GO:0019866	20	120	0.0062621	organelle inner membrane	CC
GO:0044237	130	1593	0.0062621	cellular metabolic process	BP
GO:0009059	49	438	0.0069398	macromolecule biosynthetic process	BP
GO:0016829	15	87	0.0081098	lyase activity	MF
GO:0055086	21	145	0.0082457	nucleobase-containing small molecule metabolic process	BP
GO:0006807	122	1491	0.0088455	nitrogen compound metabolic process	BP
GO:0071704	149	1919	0.0120220	organic substance metabolic process	BP
GO:1901293	15	90	0.0120263	nucleoside phosphate biosynthetic process	BP
GO:0034641	86	963	0.0137447	cellular nitrogen compound metabolic process	BP
GO:0006753	17	116	0.0251278	nucleoside phosphate metabolic process	BP
GO:0009165	14	86	0.0251278	nucleotide biosynthetic process	BP
GO:0009987	173	2338	0.0357270	cellular process	BP
GO:0005742	4	7	0.0388542	mitochondrial outer membrane translocase complex	CC
GO:0098799	4	7	0.0388542	outer mitochondrial membrane protein complex	CC
GO:0003674	233	3374	0.0405788	molecular_function	MF
GO:0006457	16	103	0.0405788	protein folding	BP
GO:0009117	16	111	0.0407647	nucleotide metabolic process	BP
GO:0020022	9	43	0.0459294	acidocalcisome	CC
GO:0051082	12	68	0.0468482	unfolded protein binding	MF
GO:0006793	42	439	0.0496207	phosphorus metabolic process	BP

Under-represented GO terms:

category	num_in_subset	num_total	adj_pval	term	ontology
GO:0005856	14	693	0.0006969	cytoskeleton	CC
GO:0015630	3	344	0.0019184	microtubule cytoskeleton	CC

e) Blue enrichment (614 genes)

Under-represented GO terms:

category	num_in_subset	num_total	adj_pval	term	ontology
GO:0006412	1	243	0.0000000	translation	BP
GO:0043043	1	245	0.0000000	peptide biosynthetic process	BP
GO:0043604	2	259	0.0000009	amide biosynthetic process	BP
GO:0006518	2	252	0.0000010	peptide metabolic process	BP
GO:0043603	3	268	0.0000025	cellular amide metabolic process	BP
GO:0003735	0	161	0.0000047	structural constituent of ribosome	MF
GO:1901566	9	381	0.0000100	organonitrogen compound biosynthetic process	BP
GO:0044271	14	476	0.0001263	cellular nitrogen compound biosynthetic process	BP
GO:1901576	27	651	0.0032823	organic substance biosynthetic process	BP
GO:0009058	29	673	0.0042627	biosynthetic process	BP
GO:0034641	47	963	0.0046486	cellular nitrogen compound metabolic process	BP
GO:0044249	28	648	0.0052861	cellular biosynthetic process	BP
GO:0009059	17	438	0.0070249	macromolecule biosynthetic process	BP
GO:0005730	9	307	0.0075215	nucleolus	CC
GO:0034645	17	433	0.0075215	cellular macromolecule biosynthetic process	BP
GO:0010467	47	891	0.0157773	gene expression	BP
GO:0003723	13	392	0.0220840	RNA binding	MF
GO:0005198	6	190	0.0280443	structural molecule activity	MF

f) Green enrichment (528 genes)

Under-represented GO terms:

category	num_in_subset	num_total	adj_pval	term	ontology
GO:0043228	56	1305	0.005741	non-membrane-bounded organelle	CC
GO:0043232	56	1305	0.005741	intracellular non-membrane-bounded organelle	CC

g) Turquoise enrichment (732 genes)

Over-represented GO terms:

category	num_in_subset	num_total	adj_pval	term	ontology
GO:0009986	15	30	0.0002845	cell surface	CC
GO:0016020	121	812	0.0002845	membrane	CC
GO:0045121	9	11	0.0002845	membrane raft	CC
GO:0098589	9	11	0.0002845	membrane region	CC
GO:0098857	9	11	0.0002845	membrane microdomain	CC
GO:0016021	60	394	0.0169210	integral component of membrane	CC
GO:0031224	60	394	0.0169210	intrinsic component of membrane	CC

Under-represented GO terms:

category	num_in_subset	num_total	adj_pval	term	ontology
GO:0043228	66	1305	0.0000000	non-membrane-bounded organelle	CC
GO:0043232	66	1305	0.0000000	intracellular non-membrane-bounded organelle	CC
GO:0005730	13	307	0.0384650	nucleolus	CC
GO:0003735	6	161	0.0498993	structural constituent of ribosome	MF
GO:0005198	8	190	0.0498993	structural molecule activity	MF

GO:0009987	179	2338	0.0498993	cellular process	BP

h) Purple enrichment (251 genes)

Over-represented GO terms:

category	num_in_subset	num_total	adj_pval	term	ontology
GO:0098798	34	240	0.0000008	mitochondrial protein complex	CC
GO:0005840	33	260	0.0001939	ribosome	CC
GO:0000313	17	120	0.0122948	organellar ribosome	CC
GO:0005743	17	120	0.0122948	mitochondrial inner membrane	CC
GO:0019866	17	120	0.0122948	organelle inner membrane	CC
GO:0005761	16	114	0.0132366	mitochondrial ribosome	CC
GO:0003735	21	161	0.0192636	structural constituent of ribosome	MF
GO:0005759	17	136	0.0192636	mitochondrial matrix	CC
GO:0005762	12	70	0.0192636	mitochondrial large ribosomal subunit	CC
GO:0044391	18	142	0.0192636	ribosomal subunit	CC
GO:0000315	12	72	0.0193209	organellar large ribosomal subunit	CC
GO:0005739	81	1359	0.0193209	mitochondrion	CC
GO:1990904	21	192	0.0193209	ribonucleoprotein complex	CC

i) Darkturquoise enrichment (100 genes)

Over-represented GO terms:

category	num_in_subset	num_total	adj_pval	term	ontology
GO:0016772	17	329	0.0218345	transferase activity, transferring phosphorus-containing groups	MF

j) Salmon enrichment (230 genes)

Over-represented GO terms:

category	num_in_subset	num_total	adj_pval	term	ontology
GO:0003723	33	392	0.0020417	RNA binding	MF
GO:0003676	45	674	0.0266022	nucleic acid binding	MF
GO:0016070	31	423	0.0266022	RNA metabolic process	BP

k) Brown enrichment (547 genes)

Over-represented GO terms:

category	num_in_subset	num_total	adj_pval	term	ontology
GO:0004016	10	43	0.0101522	adenylate cyclase activity	MF
GO:0009975	10	43	0.0101522	cyclase activity	MF
GO:0009187	10	52	0.0271632	cyclic nucleotide metabolic process	BP
GO:0009190	10	52	0.0271632	cyclic nucleotide biosynthetic process	BP
GO:0016849	10	53	0.0288921	phosphorus-oxygen lyase activity	MF

l) Pink enrichment (383 genes)

Over-represented GO terms:

category	num_in_subset	num_total	adj_pval	term	ontology
GO:0070069	5	8	0.0475195	cytochrome complex	CC

m) Tan enrichment (243 genes)

Over-represented GO terms:

category	num_in_subset	num_total	adj_pval	term	ontology
GO:0000922	5	13	0.0485818	spindle pole	CC
GO:0015630	25	344	0.0485818	microtubule cytoskeleton	CC

n) Red enrichment (460 genes)

Over-represented GO terms:

category	num_in_subset	num_total	adj_pval	term	ontology
GO:0005856	74	693	0.0113032	cytoskeleton	CC
GO:0006260	14	57	0.0284430	DNA replication	BP
GO:0005815	31	231	0.0492542	microtubule organizing center	CC
GO:0005929	127	1558	0.0492542	cilium	CC
GO:0042995	127	1558	0.0492542	cell projection	CC
GO:0051276	15	74	0.0492542	chromosome organization	BP
GO:0120025	127	1558	0.0492542	plasma membrane bounded cell projection	CC

Total enriched GO terms

Total: 171 (Total -log10(adj.pval) = 540.546811)
