# Supplementary material for: Gene co-expression network analysis of Trypanosoma brucei in tsetse fly vector
Source: Parasit Vectors. 2021 Jan 22;14:74. doi: 10.1186/s13071-021-04597-6 (PMC7821691; doi:10.1186/s13071-021-04597-6)
Supplement: Supplementary file 8 — Additional file 8: Table S4. Module KEGG enrichment results; 7 modules with their significantly over- and under-represented KEGG terms are tabulated [file 13071_2021_4597_MOESM8_ESM.pdf]

Table S4: Module KEGG Enrichment

a) Lightyellow enrichment (129 genes)

Over-represented KEGG pathway:

category	num_in_subset	num_total	adj_pval	name
path:tbr03010	26	134	1e-07	Ribosome

b) Lightcyan enrichment (193 genes)

Under-represented KEGG pathway:

category	num_in_subset	num_total	adj_pval	name
path:tbr01100	2	378	0.0078292	Metabolic pathways

c) Magenta enrichment (264 genes)

Over-represented KEGG pathway:

category	num_in_subset	num_total	adj_pval	name
path:tbr00230	12	82	0.0088518	Purine metabolism

d) Blue enrichment (614 genes)

Under-represented KEGG pathway:

category	num_in_subset	num_total	adj_pval	name

path:tbr03010	1	134	0.0143821	Ribosome

e) Red enrichment (460 genes)

Over-represented KEGG pathway:

category	num_in_subset	num_total	adj_pval	name
path:tbr03440	6	14	0.0148137	Homologous recombination

f) Yellow enrichment (539 genes)

Over-represented KEGG pathway:

category	num_in_subset	num_total	adj_pval	name
path:tbr04144	8	35	0.0249234	Endocytosis

g) Pink enrichment (383 genes)

Over-represented KEGG pathway:

category	num_in_subset	num_total	adj_pval	name
path:tbr03013	8	42	0.0318986	RNA transport

Total enriched KEGG pathway

Total: 7 (Total -log10(adj.pval) = 17.970866)
